# Global diversity and distribution of prophages are lineage-specific within the *Ralstonia solanacearum* species complex

**DOI:** 10.1186/s12864-022-08909-7

**Published:** 2022-10-06

**Authors:** Samuel T. E. Greenrod, Martina Stoycheva, John Elphinstone, Ville-Petri Friman

**Affiliations:** 1 Department of Biology, University of York, York, UK; 2 Fera Science Ltd, National Agri-Food Innovation Campus, Sand Hutton, York, UK

**Keywords:** *Ralstonia solanacearum*, RSSC, Prophage, Plant pathogenic bacterium, Mobile genetic element, Coevolution, Diversity

## Abstract

**Background:**

*Ralstonia solanacearum* species complex (RSSC) strains are destructive plant pathogenic bacteria and the causative agents of bacterial wilt disease, infecting over 200 plant species worldwide. In addition to chromosomal genes, their virulence is mediated by mobile genetic elements including integrated DNA of bacteriophages, *i.e.*, prophages, which may carry fitness-associated auxiliary genes or modulate host gene expression. Although experimental studies have characterised several prophages that shape RSSC virulence, the global diversity, distribution, and wider functional gene content of RSSC prophages are unknown. In this study, prophages were identified in a diverse collection of 192 RSSC draft genome assemblies originating from six continents.

**Results:**

Prophages were identified bioinformatically and their diversity investigated using genetic distance measures, gene content, GC, and total length. Prophage distributions were characterised using metadata on RSSC strain geographic origin and lineage classification (phylotypes), and their functional gene content was assessed by identifying putative prophage-encoded auxiliary genes. In total, 313 intact prophages were identified, forming ten genetically distinct clusters. These included six prophage clusters with similarity to the *Inoviridae*, *Myoviridae*, and *Siphoviridae* phage families, and four uncharacterised clusters, possibly representing novel, previously undescribed phages. The prophages had broad geographical distributions, being present across multiple continents. However, they were generally host phylogenetic lineage-specific, and overall, prophage diversity was proportional to the genetic diversity of their hosts. The prophages contained many auxiliary genes involved in metabolism and virulence of both phage and bacteria.

**Conclusions:**

Our results show that while RSSC prophages are highly diverse globally, they make lineage-specific contributions to the RSSC accessory genome, which could have resulted from shared coevolutionary history.

**Supplementary Information:**

The online version contains supplementary material available at 10.1186/s12864-022-08909-7.

## Background

Bacteriophages, or phages for short, are viruses that infect bacteria. They outnumber bacteria by up to six orders of magnitude [1] and through mutualistic and antagonistic interactions are thought to have a significant impact on bacterial population dynamics and evolution [2, 3]. While some phages are obligately lytic, killing their hosts after successful infection [4], other phages have a lysogenic life cycle where phage genetic material integrates into the host chromosome forming a prophage. Lytic phages can impose strong bottom-up density regulation of bacteria across different ecosystems, driving nutrient turnover by infecting their host bacteria [5, 6]. They can also drive bacterial diversification through frequency-dependent selection [5], whilst selecting for phage resistance evolution via the acquisition of phage defence systems [7] and cell membrane alterations that disrupt phage infection [8]. In contrast, temperate prophages tend to have a more modest effect on bacterial population dynamics as they often replicate during bacterial cell division and become lytic only when induced by environmental stresses such as UV irradiation or antibiotic treatment [9, 10]. Temperate phages can drive bacterial evolution by facilitating the lateral transfer of auxiliary genes which are expressed in the prophage state [11]. These genes are associated with bacterial antibiotic resistance [12, 13], competitiveness [14], and virulence [15, 16], being responsible for the disease severity of important bacterial pathogens including shigatoxigenic *Escherichia coli* [17] and *Vibrio cholerae* [18]. Temperate phages can also affect host fitness by changing gene expression or knocking out host genes after inserting into the genome [19], providing resistance to secondary phage infection, termed “superinfection immunity” [20], and by acting as hotspots of recombination [21]. Prophages are thus often beneficial for their host bacteria and are overrepresented in the genomes of pathogenic bacteria [15].

While prophages have been studied extensively with human opportunistic bacteria, they are also important for the fitness and evolution of plant pathogenic bacteria [16, 22]. For example, prophages of some of the most destructive plant pathogens including *Pseudomonas* [23], *Xylella* [24], and *Xanthomonas* [25] spp., have been associated with auxiliary genes that encode plant immune response inhibitors [26, 27], secretion system proteins, degradative enzymes, and toxin exporters [28, 29, 30]. Plant pathogen competitiveness can also be mediated by prophages that encode competitor-repressing bacteriocins [31], provide resistance to environmental stresses such as toxic metal ions [32] and antimicrobials [33], or encourage survival during nutrient scarcity by increasing metabolic potential [34]. However, the distribution, diversity, and functional potential of prophages are still relatively understudied at the pangenome-level with plant pathogenic bacteria. Prophage pangenome studies in plant pathogenic bacteria have primarily been carried out with *Dickeya* spp. and *Pectobacterium* spp. from the soft rot *Pectobacteriaceae* family. These studies have focused on prophage diversity and auxiliary gene content, identifying prophage clusters harbouring bacterial genes involved in ecological fitness and virulence [32, 35, 36]. Notably, whilst prophages are generally only present in approximately half of *Pectobacteriaceae* strains, nearly all prophages contain fitness-associated bacterial ORFs [32, 35, 36]. Subsequently, variability in prophage presence, movement, and auxiliary gene content has been linked to variation in virulence, swimming motility, and cellulase production [35]. By analysing multiple bacterial strains, these studies have provided new insights into prophage diversity and suggest that prophages may be fundamental drivers of phenotypic diversity in plant pathogenic bacterial populations. They have also shed some light on whether certain prophages can be considered to belong to the core (shared by all host strains) or accessory (shared only by a subset of strains) genomes of their hosts. However, our understanding is still based on a relatively small number of genomes [32, 35, 36] derived either from local [35] or publicly available databases [32, 36, 37], which are often susceptible to sampling biases. As a result, the wider diversity, distribution, and auxiliary gene content of prophages in plant pathogenic bacteria are likely underestimated.

*Ralstonia solanacearum* species complex (RSSC) is a genetically diverse group of plant pathogenic bacteria and is a causative agent of bacterial wilt [38] with a broad host range of over 200 plant species within 50 families [39, 40]. RSSC strains are classified into four lineages, termed phylotypes [41, 42], which generally follow their geographical location of isolation: Phylotype I includes strains originating primarily from Asia, Phylotype II from America, Phylotype III from Africa and surrounding islands in the Indian ocean, and Phylotype IV from Indonesia, Japan, and Australia [41]. Recently, the four phylotypes have been redefined as three separate species, including *R. solanacearum *sensu stricto (Phylotype II), *R. pseudosolanacearum* (Phylotypes I and III) and an array of *R. syzygii* subspecies (Phylotype IV) [43]. Considerable variation exists between and within RSSC lineages regarding their metabolic versatility [44] and tolerance to environmental stresses, including starvation and low temperatures [45, 46]. RSSC virulence and competitiveness are largely determined by a diverse accessory genome, including many prophages from the *Inoviridae* and *Myoviridae* families which are known to have a direct influence on pathogen virulence. For example, temperate phages of the *Inoviridae* family, within two clades labelled RSS-type and RSM-type, can differentially affect host virulence by up or downregulating the expression of virulence factors. The RSS-RSM intermediate φRS551 can also increase host bacterium competitiveness [31], possibly by increasing metabolic versatility via virulence factor down-regulation [47]. In contrast to *Inoviridae* phages, infection with *Myoviridae* phages, such as φRSA1, φRsoM1USA, and φRSY1, appears to have a limited impact on host virulence [48, 49, 50], despite φRSY1 lysogens showing increased twitching motility and aggregation frequency [49], typically indicative of enhanced virulence [51]. Recently, RSSC prophage diversity was assessed through an analysis of 120 *Ralstonia* spp. genomes available in the NCBI database [37]. This study revealed many characterised and novel prophages, the latter of which belonged to either the virulence-associated *Inoviridae* family or had no similarity to known phages. It also highlighted prophage-encoded auxiliary genes with potential roles in host virulence, cellular metabolism, environmental stress tolerance, and antibiotic resistance. However, while this made a significant contribution to understanding RSSC prophages using publicly available RSSC genomes [37], it had a sampling bias with low representation of strains originating from Africa and Europe, missing a subset of hosts which are cold-adapted [45].

Here, we build upon the work of Goncalves et al. 2021 [37] by conducting a comprehensive pangenome analysis of prophages within the RSSC using a new, representative collection of 192 RSSC draft genome sequences. These included isolates from all four phylotypes and six continents with extensive sampling from Africa and Europe. We specifically aimed to: i) characterise the global diversity and distribution of prophages in RSSC; ii) assess whether prophages are spread throughout their host phylogeny or show host lineage-specific distributions; and iii) investigate prophage auxiliary gene content. Intact prophages were identified in 88% of isolates and formed ten genetically distinct clusters, four of which had no similarity to known phages and so may represent novel phage groups. Prophage clusters and sub-groups had broad geographical distributions but were primarily found within specific RSSC lineages. In addition, genetically similar bacterial hosts were found to have similar prophage contents. Finally, prophages were found to contain many genes involved in bacterial and phage metabolism and virulence. By using a new, representative RSSC genome collection, this analysis provides a novel insight into the diversity and distribution of RSSC prophages as well as their contribution to the RSSC accessory genome.

## Methods

### RSSC sequencing and genome assembly

RSSC prophage hosts were selected from Protect and the National Collection of Plant Pathogenic Bacteria (NCPPB) and other reference strains maintained at Fera Science Ltd. Genomic DNA extraction was performed on 384 isolates using Qiagen DNeasy Blood and Tissue Kit (DNeasy® Blood & Tissue Handbook, Qiagen, Hilden, Germany, 2020) followed by quantification of double stranded DNA products using Quantit dsDNA Assay Kit Broad range and Nanodrop (Thermo Fisher Scientific, Waltham, MA, USA). Bacterial DNA was sequenced using Illumina MiSeq at the Earlham Institute, UK. Sequence read quality was assessed using FastQC [52] and trimming of adapters and low-quality ends was performed using Trimmomatic v.0.39 [53]. Reads were then assembled into draft assemblies using Unicycler v.0.4.8 on strict mode [54]. To classify the genomes, a pangenome analysis was performed on 192 high quality genome assemblies (Table S1) to identify core genes using *R. picketti* 12b as an outgroup [55]. Using Panaroo v1.2.4 [56] with strict mode and MAFFT aligner [57], a core genome alignment was generated. A maximum likelihood (ML) phylogenetic tree was then constructed through a phylogenomic analysis with IQ-TREE [58] and GTR + G4 model after model selection with ModelFinder [59] and two bootstrap methods (UFBoot and SH-aLRT) to look at branch support [60]. Genomes forming clusters were assigned to phylotypes based on reference strains with known phylotype (Fig. S1; Table S2). This whole genome phylotyping approach increases the accuracy of identification relative to methods relying on a few marker genes [41] and is better suited for phylotype II RSSC isolates (especially Race 3 Biovar 2), which are highly clonal [61].

### Prophage sequence identification and filtering

Putative prophage regions were identified in RSSC draft genomes using PhiSpy v.4.2.21 [62], Virsorter2 v.2.2.3 [63] + CheckV v.0.9.0 [64], and PHASTER (PHAge Search Tool Enhanced Release) [65]. PhiSpy was run using default settings with “–phage genes” parameter set to 0 to increase prophage identification sensitivity. Virsorter2 was run using default settings with the “—min-length” parameter set to 1500 to remove short prophage hits. Virsorter2 hits were filtered to remove false hits using CheckV, with only predicted proviruses kept. PHASTER was used by uploading RSSC genomes to the PHASTER web server (https://phaster.ca/). While PhiSpy and Virsorter2 + CheckV are unable to predict prophage completeness, PHASTER hits were automatically classified into intact (score > 90), questionable (score 70–90), and incomplete (score < 70) prophages based on their sizes, similarity to known phages, and the presence of phage-like and phage cornerstone genes (for example, ‘capsid’, ‘head’, ‘plate’, ‘tail’, ‘coat’, ‘portal’ and ‘holin’).

Intact PHASTER prophages were selected for downstream analysis and were filtered to retain novel prophages while reducing false positives. Firstly, overlaps in the genome coordinates of intact PHASTER prophages and prophage hits from PhiSpy and Virsorter2 + CheckV were determined to identify prophages detected by more than one software. Secondly, the similarity of intact prophages to known phages in the NCBI Virus RefSeq database was determined using Mash and Megablast, with successful hits determined using Mash distance < 0.1 (Mash) and percent identity > 30%; query cover > 50% (Megablast) thresholds. Intact prophages were kept if they were validated by more than one tool or had significant similarity to known phages. The potential inducibility of intact prophages was verified by identifying cornerstone phage genes involved in phage particle structure, phage DNA replication, and cell lysis, in prophage genomes. Prophage gene content was determined through gene annotation using VIGA v.2.7.16 [66] with parameters E-value < e^−5^ and amino acid identity > 30%. Prophage pangenome analysis was carried out using Roary v.3.13.0 [67]. Putative cornerstone phage genes were identified manually in the prophage pangenome and were sorted into “Phage structural protein”, “Phage DNA replication and packaging”, and “Phage cell lysis” categories.

PHASTER intact prophage elements that were filtered out, or were not identified as “intact”, were considered “incomplete”. The relationships between incomplete and intact prophages were characterised using Mash, with potentially related prophages determined using a Mash distance < 0.1 threshold.

### Prophage diversity analysis

Initially, intact prophage diversity was investigated by analysing prophage gene content to search for shared phage core genes, which could have been used in phylogenetic tree construction. However, no shared core genes were identified, and so prophage diversity was instead assessed using a multifaceted, alignment-free approach (Mash) [68]. Firstly, prophage genetic distances were calculated based on the presence of shared k-mers using Mash v.2.2 [68]. Mash distance matrices were generated using the “mash triangle” command with sketch size = 10,000, with similar prophages identified using Euclidean clustering. A Mash distance neighbour-joining (NJ) tree was constructed using Mashtree v.1.2.0 [69] with default parameters. Secondly, prophage gene content profiles were compared through gene annotation and pangenome analysis, as previously described. Finally, prophage GC content and lengths were determined and compared using SeqKit v.0.12.0 [70].

Prophage taxonomic identities were determined using Mash distances and Megablast against the NCBI Virus RefSeq database, as previously described. They were further determined by downloading known RSSC phage genomes from the NCBI Virus RefSeq database and including them in the prophage Mash distance NJ tree to determine relatedness between known prophages and ones identified in this study based on their clustering.

### Determination of global prophage distribution and diversity in RSSC

Prophage global and pangenome-level distribution was assessed using metadata on RSSC host geographical origin and phylotype classification (Table S1). Phylotype classification was determined based on whole genome phylogeny clustering as described earlier. Prophage host phylotype distributions were determined by comparing prophage presence and absence against the RSSC ML tree.

The potential signal of prophage-host coevolution was assessed by comparing genetic dissimilarity between RSSC hosts with their prophage contents. Due to low sample sizes for phylotypes III and IV, this was only investigated using hosts from phylotypes I, IIA, and IIB (174/192 hosts). The RSSC ML tree was used for host dissimilarity and a Bray–Curtis dissimilarity measure was used for prophage content dissimilarity, which accounted for the presence, absence, and relative abundance of prophages in host genomes. A pairwise prophage Bray–Curtis dissimilarity matrix was generated using the R ‘vegan’ v.2.6–2 package and was used to construct a UPGMA tree with R ‘phangorn’ v.2.8.1 package. A tanglegram between the RSSC ML tree and the prophage Bray–Curtis UPGMA tree was generated with functions in the R ‘ape’ v.5.6–1 package, using the ‘phytools’ v.1.0–3 package to rotate the RSSC ML tree to minimise connected lines crossing between the trees. Congruence between the RSSC ML tree and the prophage Bray–Curtis UPGMA tree was assessed using Procrustes Approach to Cophylogenetic Analysis (PACo) [71] v.0.4.2 in R. Briefly, cophenetic distance matrices were constructed using the prophage Bray–Curtis and RSSC phylogenetic trees. The distance matrices were then compared using PACo to determine the statistical significance of tree congruence using a Procrustean super-imposition of the sum of squared 10,000 network randomizations under the “r0” randomization model.

RSSC genetic dissimilarity was further compared with prophage dissimilarity with a linear mixed model using functions in the R ‘nlme’ v.3.1–152 package. The model used average host genetic dissimilarity as predictor variable, the average prophage dissimilarity as response variable, and host phylotype as a random effect. Average RSSC genetic dissimilarity was determined by generating a pairwise Mash distance matrix of hosts within each phylotype and calculating the average pairwise Mash distance for each host. Average prophage dissimilarity was determined by generating a pairwise prophage Bray–Curtis dissimilarity matrix of hosts within each phylotype and calculating the average pairwise Bray–Curtis dissimilarity for each host. To remove non-linearity, the average RSSC genetic dissimilarity was log-transformed.

### Putative auxiliary gene analysis

Putative auxiliary genes were initially identified through manual curation of prophage annotations generated using VIGA v.2.7.16 [66] with parameters E-value < e^−5^ and amino acid identity > 30% and Roary v.3.13.0 [67]. As VIGA was unable to annotate the majority of CDS (labelled “Hypothetical protein”), all putative hypothetical proteins present in more than five prophages (96 proteins) were re-annotated based on their predicted 3-D structures using a custom Python linker tool (available at https://github.com/SamuelGreenrod/Structure-based_annotation). Briefly, 3-D structures of consensus hypothetical protein amino acid sequences were predicted using Alphafold 2.0 [72]. Hypothetical protein functions (GO terms) were then predicted based on their putative 3-D structures using DeepFRI v.1.0.0 [73], with successful hits determined using a DeepFRI score > 0.5. Tool power and accuracy was tested using known proteins from *R. pseudosolanacearum* strain GMI1000 (NCBI Genbank assembly accession: GCA_000009125.1), including respiration proteins, transporters, type III effectors, and transcriptional regulators (Table S3). Prophage auxiliary genes were further investigated through Megablast of prophage genomes against the CAZy (http://www.cazy.org/) [74], Ralsto T3E (https://iant.toulouse.inra.fr/bacteria/annotation/site/prj/T3Ev3/) [75], and PHI-base databases (http://www.phi-base.org/) [76] with parameters E-value < e^−5^. Comparisons within databases generally provided multiple overlapping hits for each putative auxiliary gene. Therefore, in all searches, only the top hit was used if multiple hits overlapped. Prophage potential disruption of type III effectors was determined by generating a circular genome map of the comprehensively annotated *R. solanacearum* UY031 strain chromosome (NCBI Reference sequence: NZ_CP012687.1) with GView [77] and mapping prophages using the GView Blast Atlas function. UY031 gene product labels were determined using the NCBI GenBank annotation. UY031 was chosen as a reference genome as it has the closest complete genome to phylotype IIB strains that only showed potential disruption of type III effectors by prophages.

### Data visualisation and statistical analysis

Statistical analyses and data visualisation were carried out using Microsoft Excel v.2102 [78], R v.4.0.3 [79] and RStudio v1.4.1103 [80]. Putative prophage genome sizes and GC content were compared using two-way ANOVA. Equal variance and normality assumptions were met using Box-Cox transformation. Comparisons of intact prophage number, RSSC genetic dissimilarity, and prophage dissimilarity between phylotypes were compared using Kruskal–Wallis tests (with Dunn’s test for pairwise comparisons) and linear regression. Graphs and heatmaps were made using the R ‘ggplot2’ v.3.3.3 and R ‘pheatmap’ v.1.0.12 packages, respectively. Venn diagrams were made using http://www.interactivenn.net/ and Inkscape v1.1.1 [81]. The Mash NJ and ML phylogenetic trees were visualised using the R ‘ggtree’ package v.2.1.4. The world map in Fig. 4 was generated using the R ‘maps’ package v.3.4.0 and was derived from the Natural Earth project (https://www.naturalearthdata.com/), which is open access (https://www.naturalearthdata.com/about/terms-of-use/).

## Results

### Prophages are commonly found in RSSC genomes

Prophages were identified using PhiSpy, Virsorter2 + CheckV, and PHASTER, in 192 RSSC hosts sampled from six continents, representing phylotypes I (56), IIA (22), IIB (96), III (8), and IV (10) (Table S1). In total, 1,318 prophage regions were identified across all hosts, including 400 with PhiSpy, 380 with Virsorter2 + CheckV, and 538 with PHASTER (Table S4A-D; Fig. S2A). PHASTER prophage regions included 344 intact prophages, 73 questionable regions, and 121 incomplete prophages as determined based on PHASTER criteria [65]. 758 prophage hits were identified by more than one software and so prophage hits were de-duplicated leaving 878 unique putative prophage elements (Table S4E). PHASTER intact prophages, which are predicted to be inducible, were filtered to remove false positives by comparing their predictions with other tools and determining their taxonomic identities based on similarity to known phages in the NCBI Virus RefSeq database. 222/344 (64.5%) intact prophages were identified with more than one tool and 184/344 (53.5%) could be assigned taxonomic classifications (Fig. S2B). Combined, 313/344 (91.0%) intact prophages either had tool cross-validation or significant similarity to known phages (Table S4F). Filtered intact prophages were further assessed for potential inducibility based on the presence of cornerstone phage genes involved in phage particle structure, phage DNA replication and packaging, and phage cell lysis. All filtered intact prophages contained genes belonging to at least two of the categories (Fig. S2C) and so were used in the analysis. Intact PHASTER hits that were removed through filtering or PHASTER hits which had “Questionable” or “Incomplete” labels were considered putative incomplete prophages.

**Fig. 1:**
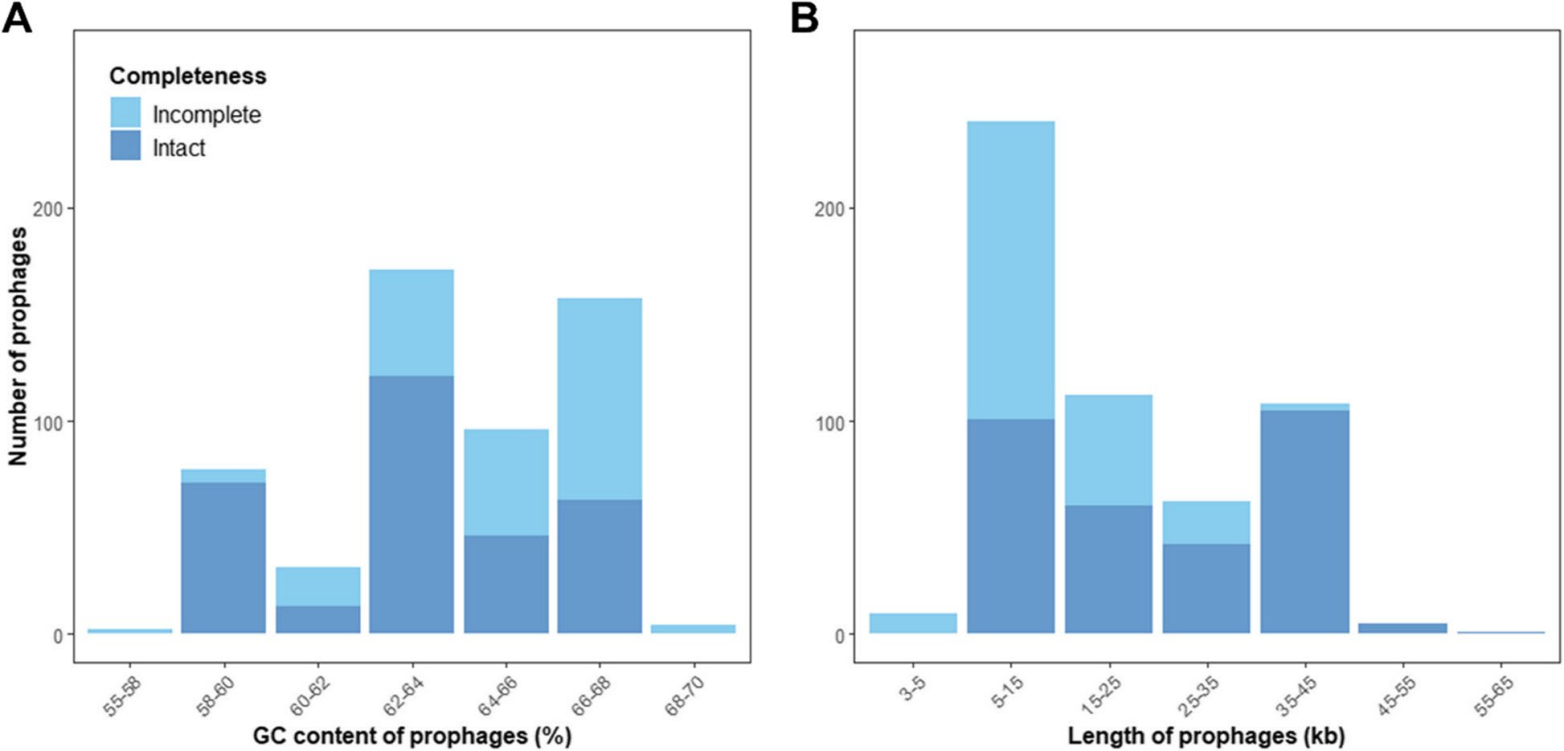
RSSC prophages have a multimodal length and GC content distribution. (**a**) GC content and (**b**) length distributions of validated intact and putative incomplete RSSC prophages. Bars are coloured by putative prophage completeness

Intact prophages were found in 169/192 host genomes (88.0%). Intact prophages had similar GC content to related incomplete prophages (ANOVA: F = 0.111; d.f = 1, 115; *p* = 0.74) (Fig. 1A; Fig. S3A). However, intact prophages had significantly greater genome sizes than related incomplete prophages (ANOVA: F = 56.69; d.f = 1, 115; *p* < 0.001) (Fig. 1B; Fig. S3B). Prophage GC content and length distributions were multimodal, containing three and two peaks, respectively, possibly indicating that RSSC prophages include genetically distinct groups. Polylysogeny, where more than one intact prophage is integrated into a genome, was found in 124 hosts (64.6%). The average number of putative prophage regions per genome was 2.8 ± 1.42 s.d, while the number of intact prophages per genome was 1.64 ± 0.85. The number of intact prophages per genome was significantly different between phylotypes (Kruskal–Wallis: x^2^ = 36.4; d.f = 4, *p* < 0.05). However, pairwise comparisons using Dunn’s test suggested that only one comparison was significantly different with phylotype IIB having more prophages per genome than phylotype IIA (Fig. S4, *p* < 0.05). Similarly, the number of incomplete prophages was significantly different between phylotypes (Kruskal–Wallis: x^2^ = 91.7; d.f = 4, *p* < 0.001), with significant differences only found between phylotype IIB and other phylotypes (*p* < 0.05).

### RSSC genomes contain ten genetically distinct prophage clusters

**Fig. 2:**
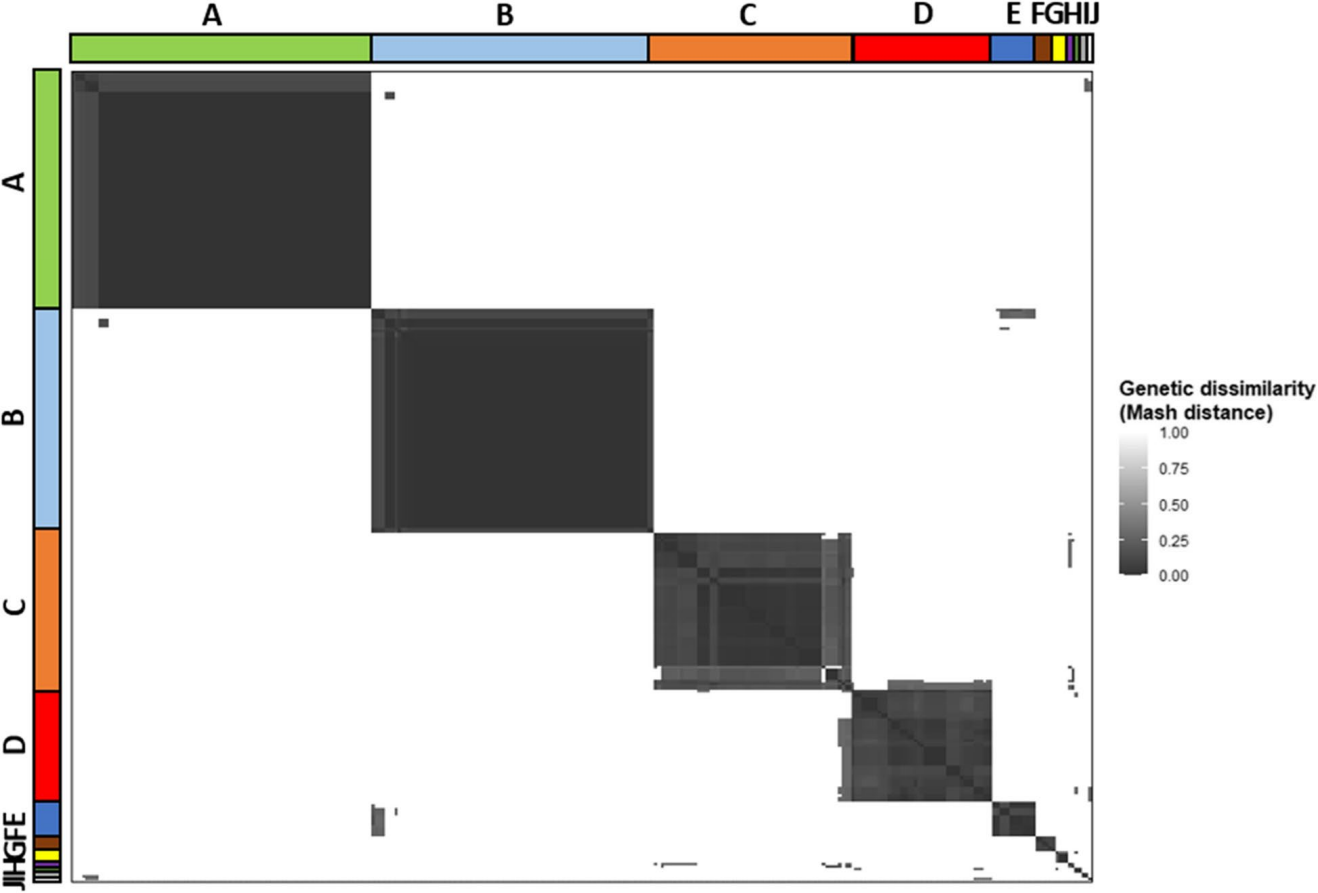
Prophages are clustered into ten separate groups and one singleton. Pairwise Mash distance heatmap of RSSC prophages, clustered using Euclidean distances. Clusters are labelled with both letters (**A**-**J**) and coloured bands. Prophage similarity (dark grey) decreases with increasing Mash distances (white)

**Fig. 3:**
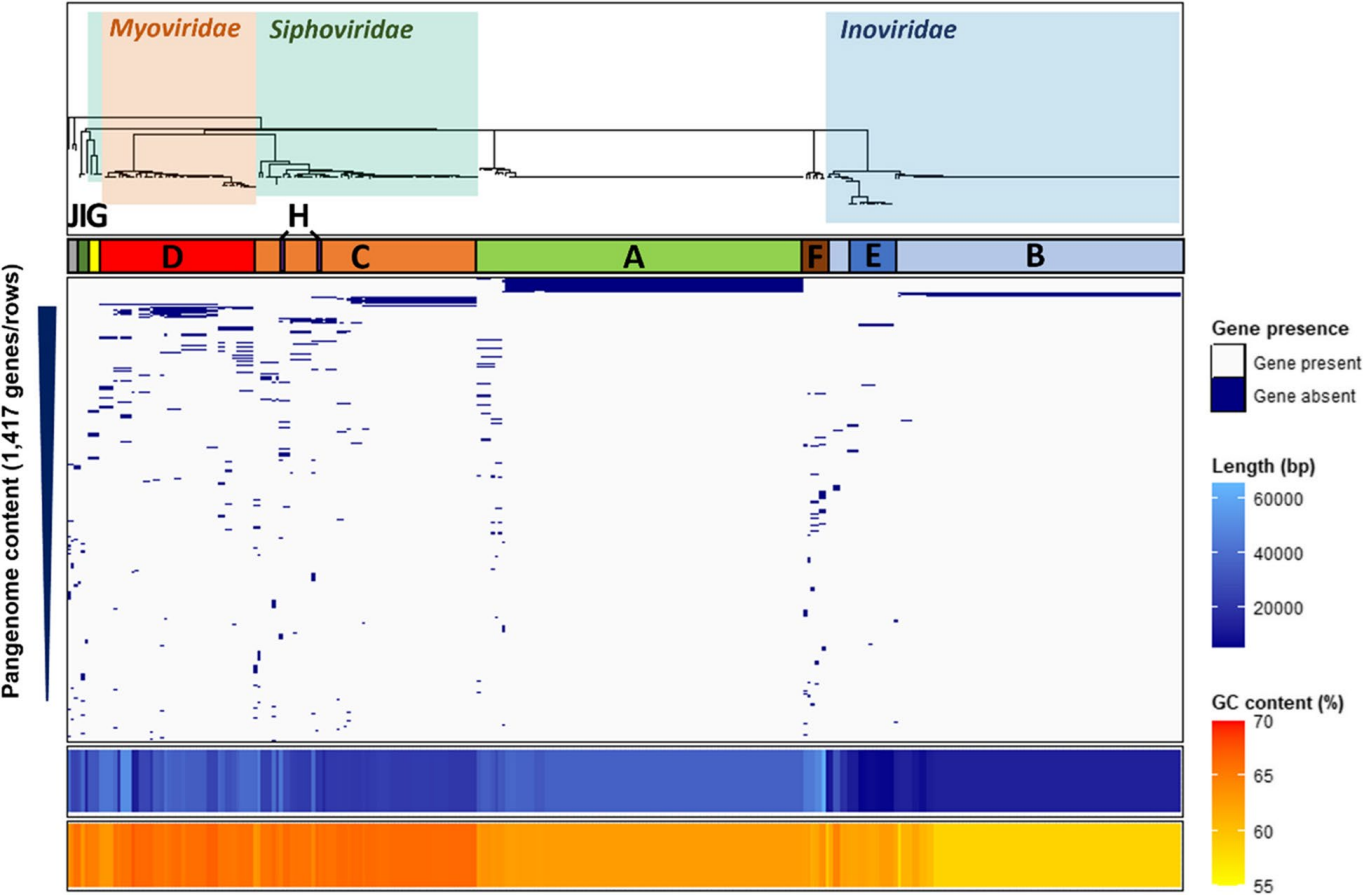
Prophage clusters show unique gene content, GC content, and length profiles. From top-down: Prophage Mash distance neighbour-joining tree (see Fig. S5), coloured and labelled with phage families. Letters and coloured bars refer to prophage clusters from Fig. 2. Blue and grey panel shows the presence (blue) and absence (grey) of genes within the prophage pangenome, shown as rows in order of gene prevalence. Bottom panels show prophage length (blue) and GC content (orange)

Due to mosaic genome architecture, the diversity and similarity of intact prophages was assessed by calculating prophage genetic distances based on the presence of shared k-mers using Mash, where genetically similar prophages were expected to have lower Mash distances. RSSC prophages formed ten clusters (labelled A-J and one singleton, Fig. 2), each representing genetically similar phage groups. Two clusters, A and B, were largely clonal, predominantly containing prophages with Mash distances equalling zero. However, the remaining clusters were more diverse, containing prophages with a range of Mash distances. Cluster E had low Mash distances with certain prophages in cluster B, indicative of potential gene exchange or divergence from a common ancestor. Similarly, cluster H had low Mash distances with prophages in cluster C. Prophage clusters were further visualised using a Mash distance neighbour-joining tree, which supported the clusters identified with Euclidean distances, and were further verified by comparing their gene contents, GC contents, and lengths (Fig. 3). Prophage pangenome content was determined through gene annotation, identifying a total of 1,417 unique genes. Each prophage cluster had a unique gene content profile (Fig. 3), containing between 74–100% cluster-specific genes (Table 1). However, no core prophage genes (found in > 90% of isolates) were identified. Most prophage clusters also had distinct GC content and length boundaries (Fig. 3; Table 1), except for cluster D which had high gene content, GC content, and length variation.

**Table 1:** Genetic characteristics of identified prophage clusters

Prophage cluster	Proportion of genes that are cluster-specific	Average GC content (% ± sd)	Average length (kbp ± sd)	Taxonomic classification
A	162/164 (99%)	62.9 ± 0.18	35.2 ± 1.3	Uncharacterised
B	74/76 (97%)	59.2 ± 1.4	13.3 ± 3.1	*Inoviridae*
C	272/298 (91%)	66.1 ± 0.54	22.9 ± 4.2	*Siphoviridae*
D	316/316 (100%)	65.8 ± 0.93	33.2 ± 9.3	*Myoviridae*
E	31/31 (100%)	62.4 ± 0.35	7.5 ± 2.2	*Inoviridae*
F	205/205 (100%)	62.7 ± 0.77	48.4 ± 6.6	Uncharacterised
G	53/53 (100%)	65.1 ± 0.30	27.7 ± 8.3	*Siphoviridae*
H	75/101 (74%)	65.1 ± 1.8	42.4 ± 3.3	*Siphoviridae*
I	45/45 (100%)	66.0 ± 0.050	26.4 ± 1.8	Uncharacterised
J	85/85 (100%)	64.3 ± 0.74	31.7 ± 8.9	Uncharacterised

### Prophage clusters include both known and potentially novel, uncharacterised phages

The taxonomic identities of the prophage clusters were determined using the NCBI Virus RefSeq database. A total of 211 prophages (67.4%) from six clusters (B, C, D, E, G, H) had similarity to the *Inoviridae*, *Myoviridae, and Siphoviridae* phage families within the *Caudovirales* order (Fig. 3; Table S4F). Clusters B and E represented the *Inoviridae* family; Cluster B contained the RSM-type phages with a large clonal group identified as φRS551, and a smaller group identified as φRSM3. Cluster E contained RSS-type prophages identified as φRSS1, φRSS30, φRSS-TH1, in addition to φPE226. Cluster D represented the *Myoviridae* family containing prophages identified as φRSA1, φRSY1, and φRsoM1USA. Clusters C, G, and H represented the *Siphoviridae* family; Clusters C and H contained a prophage identified as φDina while cluster G contained φRS138. Known RSSC temperate phages aggregated with clusters in the same families (Fig. S5), supporting prophage identity determination. The remaining 102 prophages (32.6%) from four clusters (A, F, I, J) had no sequence similarity with phages present in the NCBI Virus RefSeq database (last accessed July 2022) and showed no clear clustering with any known phages. Therefore, these clusters may represent novel, uncharacterised prophage groups.

### Prophages have broad geographical distributions reflecting the host evolutionary history

**Fig. 4:**
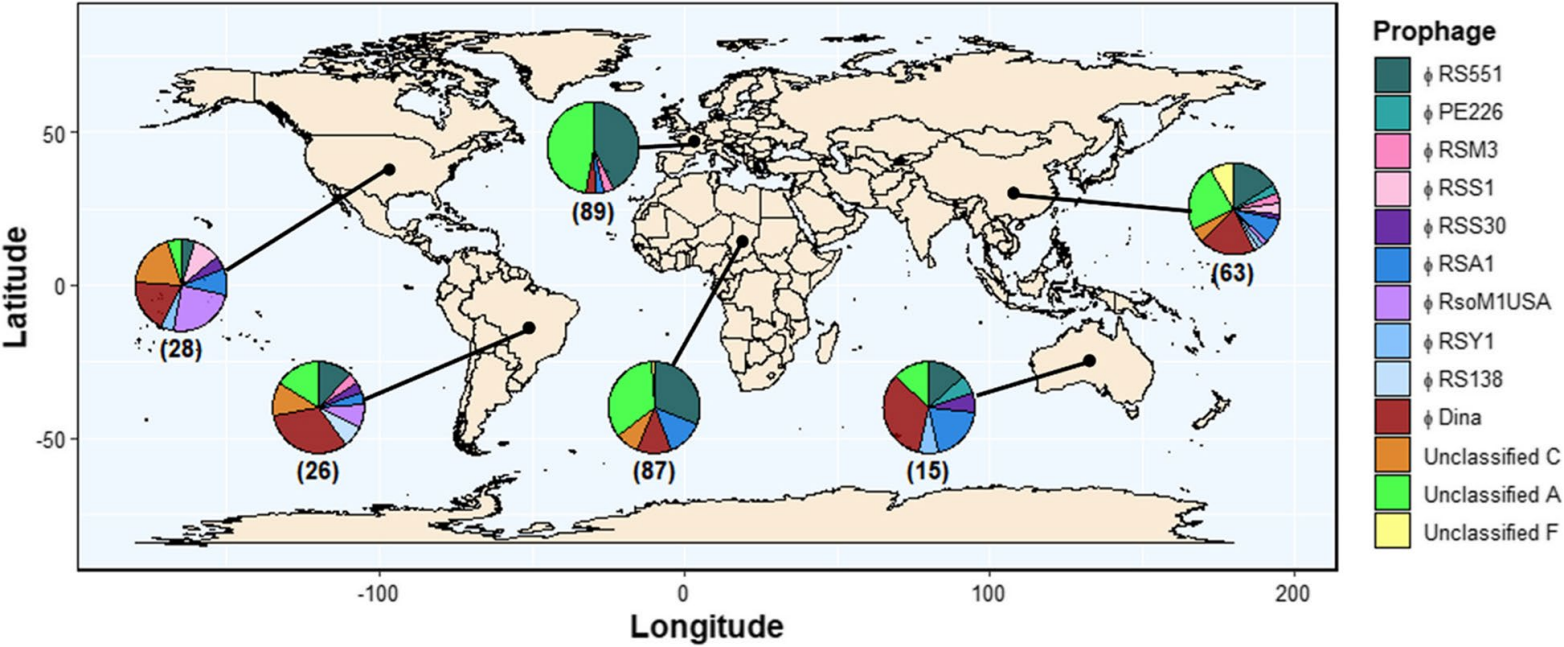
Prophages have broad geographical distributions. World map overlaid with pie charts of prophage relative abundances in six continents (from left to right: North America, South America, Europe, Africa, Oceania, and Asia). To aid visualisation only prophages found in more than two isolates are shown (see Fig. S6 for all). Black lines link pie charts to continents. Numbers in brackets refer to the number of prophages represented by each pie chart for each continent. Map is available at https://www.naturalearthdata.com/

The global distribution of RSSC prophages was determined by assessing their presence and absence across different continents, including Africa, Asia, Europe, North America, South America, and Oceania (Fig. 4; Fig. S6). Phage families had broad geographical distributions; *Inoviridae*, *Myoviridae*, and *Siphoviridae* prophages were distributed across all six continents. Individual prophages were generally found in 2–3 continents, while some prophages were more widespread. The *Inoviridae* phage φRS551, *Myoviridae* phage φRSA1, *Siphoviridae* phage φDina, and novel prophage Unclassified A were found in all six continents. Only one low abundance prophage, φRSS-TH1, was continent-specific, being found exclusively in Asia.

Despite broad geographical distributions, prophages tended to have higher abundances in certain continents; φRS551 and Unclassified A were primarily found in hosts from Africa (φRS551, 36%; Unclassified A, 34%) and Europe (φRS551, 45%; Unclassified A, 43%), and φRSA1 and φRsoM1USA were primarily found in Africa (φRSA1, 44%) and North America (φRsoM1USA, 63%), respectively. 5/6 (83%) of Unclassified F prophages were found in Asia. Prophages were also absent from certain continents; φRSY1 and φRsoM1USA were not found in any host bacteria from Africa or Europe, whilst φDina was abundant in all continents except for Europe. Therefore, RSSC prophages appear to mainly follow continent borders.

**Fig. 5:**
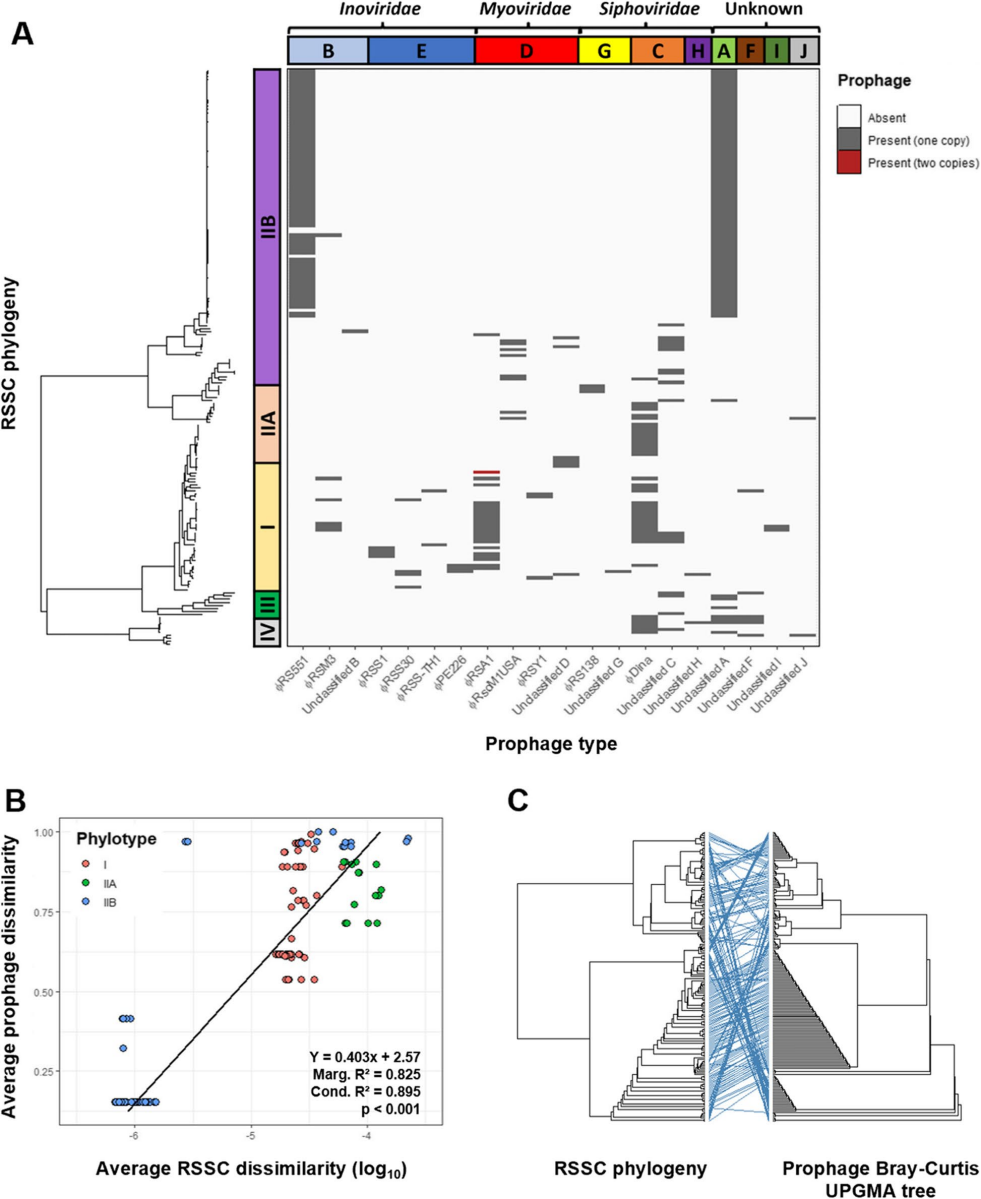
Prophages are phylotype-specific and have diversity proportional to the genetic diversity of their hosts. **a** Maximum likelihood tree of RSSC isolates from phylotypes I, IIA, IIB, III, and IV rooted and annotated with prophage presence (dark grey) and absence (white). Coloured bars on left show phylotype clustering within RSSC tree. Coloured bars on top show prophage clusters, labelled with phage families. **b** Average RSSC dissimilarity (log-transformed), measured using average pairwise Mash distances, versus average prophage dissimilarity, measured using average pairwise prophage Bray–Curtis distances. Points are coloured by phylotype. Bottom-right box shows regression equation, marginal and conditional R^2^ statistics, and *p*-value. Regression line is plotted. **c** Tanglegram of RSSC maximum likelihood tree and prophage Bray–Curtis UPGMA tree. Blue lines connect the same labels on each tree with horizontal lines supporting tree congruence and crossed lines indicating tree incongruence

As RSSC phylotypes tend to have different geographical origins [41], we investigated if the presence and absence of prophages was associated with specific RSSC phylotypes (Fig. 5A). *Inoviridae* prophages were predominantly found in phylotype I genomes, which exclusively contained φPE226, φRSS0, φRSS1, φRSS30, and 5/6 φRSM3 prophages. However, φRS551 was exclusively found in phylotype IIB hosts. Moreover, the *Myoviridae* prophages φRSY1 and φRSA1 were primarily found in phylotype I, whilst φRsoM1USA was found in phylotype IIB and IIA hosts. Incomplete copies of φRSM3 and φRSY1 had similar distributions to intact prophages, being exclusively found in phylotype I (Fig. S7). The *Siphoviridae* prophage φRS138 and its incomplete copy, were only found in phylotype IIA. Prophages within the same family tended to be mutually exclusive; only ten RSSC hosts contained multiple *Inoviridae* (three hosts), *Myoviridae* (one host), or *Siphoviridae* (six hosts) prophages. Unclassified A, similar to φRS551, was found predominantly in phylotype IIB with only low abundances in phylotypes I and IIA. However, it was also present in phylotype III (3/8) and IV (4/10) hosts and so may be more widespread. Unclassified G and I were exclusively found in phylotype I. Notably, φDina, Unclassified C, and Unclassified F were more generalist, being found more evenly distributed between phylotypes (Fig. 5A; Fig. S8). RSSC prophages therefore appear to be mainly phylotype-specific, with only a few prophages being associated with multiple host phylotypes.

### Genetically similar host bacteria are associated with similar prophage profiles

In addition to harbouring unique prophages, RSSC phylotypes also differed in their overall prophage profiles (Fig. 5A; Fig. S8). For example, phylotype I genomes contained 14 different prophage types, with 12/14 prophages being found in less than 15% of phylotype I hosts (Fig. S8) in addition to highly prevalent prophages such as φRSA1 and φDina. Phylotype IIA hosts only contained 6 prophage types but had 4/6 found in more than 15% of hosts, including φRsoM1USA, φRS138, φDina, and Unclassified C groups with relatively high abundance (Fig. S8). In contrast, Phylotype IIB hosts predominantly contained two high abundance prophages, φRS551 and Unclassified A, which were present in 79% and 83% of hosts, respectively (Fig. S8). Phylotypes III and IV only contained Unclassified prophage groups except for φDina which had a high abundance in phylotype IV.

Interestingly, the diversity of prophages within each phylotype appeared to reflect the branching on the RSSC phylogenetic tree (Fig. 5A), indicating that there may be an association between RSSC host and prophage genetic dissimilarities. We tested this by i) a linear regression and ii) congruence analysis between the RSSC phylogenetic maximum-likelihood and prophage dissimilarity UPGMA trees. In support of our hypothesis, phylotype IIB hosts had both significantly lower prophage dissimilarity (Kruskal–Wallis: x^2^ = 77.668; df = 2; *p* < 0.001) (Fig. S9A), and significantly lower genetic diversity than phylotypes IIA and I (Kruskal–Wallis: x^2^ = 100.6; df = 2; *p* < 0.001) (Fig. S9B). As a result, host genetic dissimilarity explained a significant amount of variation in prophage dissimilarity based on linear regression (*R*^2^ = 0.702, *p* < 0.001, Fig. 5B). This association was further verified by comparing the RSSC phylogenetic and prophage dissimilarity UPGMA trees, which showed significant congruence (M^2^_xy_ = 0.302, *p* < 0.001, *N* = 10,000, Fig. 5C). Therefore, prophages appear to be diverging in tandem with their hosts. Importantly, we found that tree congruence was not driven by individual clades as significant congruence was also present in phylotype I (M^2^_xy_ = 0.001, *p* < 0.001, *N* = 10,000), IIA (M^2^_xy_ = 0.002 *p* = 0.011, *N* = 10,000), and IIB strains (M^2^_xy_ = 0.001, *p* < 0.001, *N* = 10,000) when tested independently. These results suggest that genetically similar hosts have more similar prophage profiles, possibly reflecting a shared coevolutionary history or local adaptation.

### Prophages encode various putative auxiliary genes linked with metabolism and virulence

**Fig. 6:**
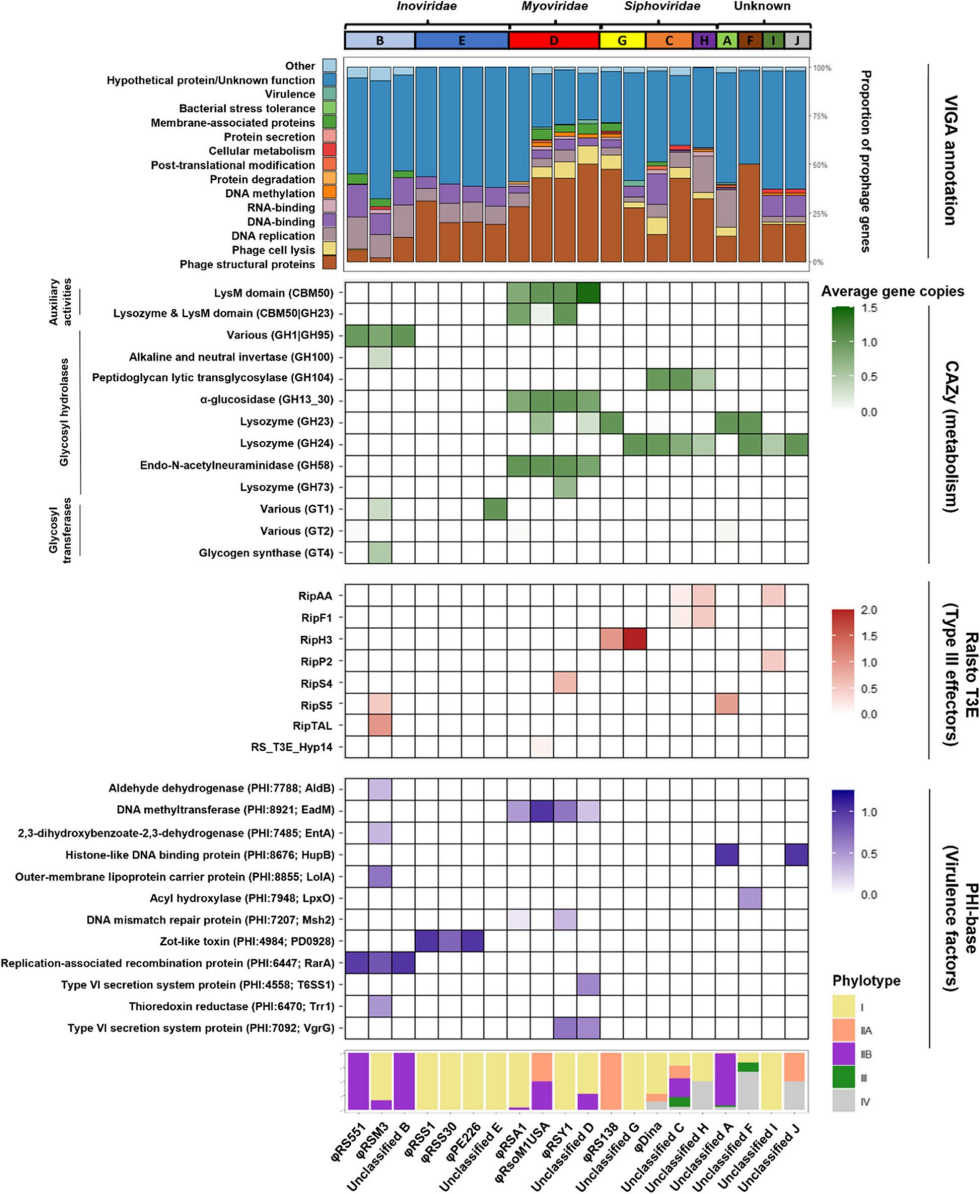
Prophages encode many putative auxiliary genes involved in different bacterial processes. Top bar chart: stacked bar chart showing prophage gene content as a proportion of total prophage genes, coloured by putative function. Numbers above bars represent total number of genes in each phage species. Heatmaps: putative auxiliary genes identified through comparisons of prophage CDS with bacterial metabolism and virulence databases coloured by absolute abundance. Different colours reflect different databases used (labelled on the right). Putative gene functions are labelled on the left. Bottom bar chart: stacked bar chart showing the phylotype distribution of isolates containing each prophage

Prophages are known to encode various auxiliary genes with potential benefits for bacterial fitness [15, 16, 17, 18, 19]. Therefore, prophage gene content was analysed for auxiliary genes through gene annotation using VIGA, a custom structure-based annotation tool, and comparisons with bacterial metabolic (CAZy) and virulence gene databases (Ralsto-T3E and PHI-Base). Most prophage genes encoded unannotated hypothetical proteins (57.9%) and cornerstone phage proteins related to structural components (21.9%), replication (6.4%), and lysis (4.3%) (Fig. 6; Table S5A). Prophages also contained many genes with potential auxiliary functions, which made up 9.5% of the prophage pangenome, including putative DNA-binding transcriptional regulators, DNA methyltransferases, membrane-associated proteins, and stress tolerance proteins. Moreover, several prophage genes with potential roles in bacterial metabolism and virulence were identified.

Putative auxiliary metabolic genes primarily encoded glycosyl hydrolases and glycosyl transferases (Fig. 6; Table S5B), the latter of which were exclusively found in *Inoviridae* prophages. Glycosyl hydrolases tended to be specific to phage family or phage type, with *Inoviridae* RSM-type phages exclusively encoding proteins with GH1|GH95 domains and *Myoviridae* prophages all encoding proteins containing LysM domains, as well as domains involved in glucosidase and neuraminidase proteins. However, *Siphoviridae* and novel, uncharacterised prophages all encoded proteins with lysozyme domains.

Putative virulence genes predominantly encoded type III effectors, transcriptional regulators, and membrane transporters (Fig. 6; Table S5C-D). Many of these were found in *Inoviridae* prophages with φRSM3 additionally containing the type III effectors RipS5 and RipTAL. However, the *Myoviridae* prophages φRSY1 and Unclassified D were found to contain type VI secretion system proteins and φRSY1 also contained the type III effector RipS4. Further, the *Siphoviridae* prophages φRS138 and Unclassified G were found to carry the type III effector RipH3. In the novel, uncharacterised prophages, putative virulence genes were found in all Unclassified groups. Unclassified F encoded an acyl hydroxylase and Unclassified I encoded the type III effectors RipAA and RipP2. Moreover, Unclassified A and Unclassified J, encoded a histone-like DNA binding protein, with Unclassified A also encoding the type III effector RipS5. As RipS5 is pseudogenised in phylotype IIB strains [82], which predominantly contain Unclassified A, the position of Unclassified A relative to the RipS5 coding sequence was assessed in the *R. solanacearum* strain UY031. Unclassified A was found to be flanked by two type III effector regions, one of which was annotated as skwp5, otherwise known as RipS5 [83] (Fig. S10). This suggests that, in phylotype IIB hosts, RipS5 may be disrupted by an Unclassified A prophage.

Similar to the auxiliary metabolic genes, some putative virulence genes were also phage family or phage type specific, with the *Inoviridae* RSM-type phages all encoding the replication-associated recombination protein RarA, whilst the RSS-type phages encoded a zot-like toxin. Moreover, all *Myoviridae* phages encoded a DNA methyltransferase. These genes were found in multiple phylotypes and may be under more general selection than other genes which were phage cluster specific. 

Following prophage gene annotation, the most prevalent gene was labelled “Hypothetical protein”, reflecting the limitations of available homology-based tools in prophage annotations. We attempted to address this by writing a Python linker tool which combines the protein structure prediction tool Alphafold 2.0 [72] with the structure–function annotation tool DeepFRI [73]. The tool was found to have high accuracy based on annotations of known RSSC proteins (Table S3). It successfully annotated 9/96 (9.4%) of the most abundant hypothetical proteins providing GO terms including DNA-binding (GO:0003677) and RNA binding (GO:0003723) (Table S5E). Overall, prophages appear to encode many putative auxiliary genes, with potential lineage-specific functional contributions to the RSSC accessory genome.

## Discussion

While prophages are known to affect plant pathogen fitness by mediating host growth, competitiveness, and virulence [26, 27, 28, 29, 30, 31, 32, 33, 34, 37, 84], only very little is known about their diversity and distribution at the pangenome-level. In this study, we analysed the prophage content of the plant pathogenic RSSC bacterium using a representative, global collection of new 192 draft genome assemblies. Prophages were found in all screened host genomes, forming ten genetically distinct prophage clusters. While most of these could be assigned to known prophages based on databases, no matches were found for four clusters, which could represent novel prophages. Interestingly, while prophages had broad geographical distributions, they showed phylotype-specific associations and genetically similar hosts had similar prophage profiles over the whole RSSC. Several potential auxiliary genes potentially linked to RSSC metabolism and virulence were also identified, and some of these were unique to specific prophage clusters and host phylotypes. Together, our results advance knowledge on the global RSSC prophage diversity and distribution at the pangenome level.

Putative prophage elements were identified in all hosts and included both intact and incomplete prophages, the latter of which were shorter albeit with having similar GC content to their intact copies. These incomplete prophages may represent “grounded” prophages which have lost the ability to excise themselves from the bacterial chromosome [21], becoming truncated. However, grounded prophages tend to ameliorate to their hosts’ nucleotide usage [85, 86], and so would be expected to have higher GC content than intact prophages. This suggests that some incomplete prophages may have become truncated recently, or may instead represent intact prophages that were omitted during prophage filtering. On average, RSSC hosts contained 1.6 intact prophages per genome, which is an intermediate number compared to other plant pathogens, ranging from less than one per genome in soft rot *Pectobacteriaceae* [32] to more than five in *Xylella fastidiosa* [24]. These results generally support a previous analysis of RSSC prophages which found that strains contained 1.2 intact prophages per genome [37]. However, the slightly higher average number of intact prophages in our study could reflect higher sampling of clonal phylotype IIB strains which typically contained two intact prophages.

Intact prophages had a multimodal length and GC content distribution, suggestive of multiple genetically distinct prophage groups. Indeed, ten prophage clusters were identified which had different gene content profiles, GC content, and lengths. Whilst six clusters belonged to the *Inoviridae*, *Myoviridae*, and *Siphoviridae* families, four clusters contained novel, uncharacterised prophages with no sequence similarity to known phages in public databases. These results are consistent with a previous genomic analysis of RSSC prophages [37] which identified both known and novel prophages and demonstrates that RSSC has high prophage diversity. Most of the prophages we identified, except for φRsoM1USA and φDina, were the same as those found previously. This was surprising given that previous analyses have relied on publicly available databases which predominantly contain hosts from South America and Asia, whilst we used a global genome collection representing all six continents. Therefore, most of the RSSC prophage diversity appears to be represented in South America and Asia. However, whether this is due to prophage diversity being particularly high in these continents, common descent, or these prophages being ubiquitous, remains unclear.

Prophages within the same family tended to be mutually exclusive, with intra-family polylysogeny only observed in ten hosts. This contradicts previous studies on *Myoviridae* phages which have found polylysogeny-promoting homology disruption at attachment sites [50], and the co-existence of both φRSA1 and φRSY1 prophages within the genome of *R. solanacearum* strain EJAT-1458 [49]. Polylysogeny could be inhibited by prophage-mediated superinfection immunity which represses secondary infection by similar phages [20]. Indeed, superinfection immunity has previously been observed in both *Inoviridae* and *Myoviridae* lysogens [87, 88]. Importantly, superinfection immunity could repress infection by lytic phages from the same family, potentially changing bacteria-phage population dynamics and disrupting the evolution of costly phage resistance mechanisms [8, 89]. In addition, it could also restrict the efficacy of phage therapies, where lytic phages are used to treat bacterial infections [90]. Therefore, prophage-mediated superinfection immunity should be further investigated, for example, by assessing the rates of lysogeny and cell lysis of *Inoviridae*, *Myoviridae,* and *Siphoviridae* lysogens following infection with different temperate and lytic phages.

The total geographical distribution of RSSC prophages remains unclear. This is mainly due to lack of research as only one genomic study investigating RSSC prophages has been published before this [37]. Here, we show that RSSC prophages have broad geographical distributions, each being found in multiple continents, with continent-specificity only observed in one low abundance prophage. Whilst these findings contrast with analyses by Gonҫalves et al. (2020) [37] which suggested that RSSC prophages are continent-specific, this is likely due to a previous reliance on geographically unrepresentative publicly available genomes. For example, φRS551 and φRSA1 were previously thought to be exclusive to South America and Korea, respectively, yet we found that they were present in six continents, with particularly high prevalence in Africa and Europe. Therefore, by using a more diverse, representative RSSC genome collection, our results suggest that prophages are more widespread than previously thought and are prevalent in all six sampled continents.

Despite being widespread, prophages generally followed continent borders reticent of the distinct geographical distributions found between RSSC phylotypes [41]. Yet, due to an under-representation of phylotype IIA and IIB hosts in publicly available genomes, previous analyses have not fully assessed prophage phylotype distributions. We found that prophages tended to be phylotype-specific with the *Inoviridae* and *Myoviridae* prophages almost exclusively found in phylotype I and the RSM-RSS intermediate φRS551 and novel prophage Unclassified A primarily found in phylotype IIB. These findings support Goncalves et al. (2020) [37] which primarily identified *Inoviridae* and *Myoviridae* prophages in *R. pseudosolanacearum* strains (phylotypes I and III). In addition, Goncalves et al. (2020) [37] also only found φRS551 in *R. solanacearum* strains (phylotypes IIA and IIB). Collectively, our results show strong evidence for lineage-specific associations with RSSC prophages. However, our results extend previous findings by showing that prophages tend to be RSSC phylotype-specific rather than species-specific. Previous studies have observed prophage lineage-specificity in other, primarily human, pathogens [91, 92, 93, 94, 95, 96], in addition to free living bacteria [97, 98], resulting in their investigation as potential molecular markers of bacterial genomic diversity [99, 100, 101]. Therefore, this may be a widespread phenomenon. Notably, some prophages, such as φDina, were found to be more generalist and were more evenly distributed across multiple phylotypes. φDina was recently discovered in agricultural samples from Mauritius and Reunion islands and was hypothesised to be a temperate phage [102]. These findings confirm this hypothesis and suggest that φDina is likely more widespread than previously thought. However, it is unclear whether φDina is a highly transmissible prophage that has been acquired independently by each phylotype or was acquired by an ancestral RSSC strain prior to phylotype divergence.

Prophage dissimilarity varied between phylotypes with phylotype IIB hosts generally containing similar prophage contents and phylotype I and IIA containing more dissimilar prophages. Prophage dissimilarity was found to be associated with host genetic dissimilarity with genetically similar hosts tending to harbour similar prophages. Further, the RSSC phylogenetic tree was congruent with a UPGMA tree constructed based on prophage content dissimilarity. Combined with prophage phylotype-specificity, this suggests that, historically, prophages and their hosts may have maintained a stable lineage-specific association and have coevolved in tandem. The co-occurrence of prophage and host evolution within phylotypes indicates that there may have been ancestral transmission barriers preventing prophages from moving between phylotypes. These may have been geographical barriers as the phylotypes are thought to have arisen from geographical isolation [103] although prophages were found to have widespread, often overlapping, geographical distributions. Alternatively, inter-phylotype prophage transmission may be limited by biological transmission barriers, such as phage defence systems, which are abundant in RSSC [7]. Furthermore, it is possible that prophages could encode specific auxiliary genes that lead to phylotype-specific ecological differences, reducing the likelihood of strain coexistence and horizontal movement of prophages. These hypotheses should be assessed experimentally in future studies.

Auxiliary genes were found to comprise approximately 9.5% of the prophage pangenome and were potentially involved in a variety of different functions including transcriptional regulation, DNA methylation, bacterial metabolism, and virulence. Prophage-encoded metabolic genes primarily included glycosyl hydrolases and glycosyl transferases which facilitate the degradation of carbohydrates and mediate pathogen virulence [104]. In RSSC, glycosylation of type IV pilin proteins by glycosyl transferases is required for biofilm formation and pathogenicity [105]. Glycosyl transferases were exclusively found in *Inoviridae* prophages, many of which are known to affect host virulence and competitiveness [31, 51, 106]. Although the virulence reduction by *Inoviridae* prophages is typically mediated by transcriptional regulators [31, 106], these results suggest that prophage-encoded auxiliary metabolic genes should be considered when assessing prophage effects on RSSC virulence and competitiveness.

Prophages also encoded a variety of putative virulence genes including type III effectors, transcriptional regulators, and membrane transporters. Notably, transcriptional regulators were identified in the *Inoviridae* prophages φRS551 and φRSM3, supporting experimental studies which have attributed reduced virulence (*i.e.*, hypovirulence) in φRS551 and φRSM3 lysogens to transcriptional repressors [31, 106]. Transcriptional regulators were also identified in Unclassified H, Unclassified J, and Unclassified A, indicating these previously uncharacterised prophages may also affect host virulence. Interestingly, despite reducing host virulence in experimental assays, φRSM3 was found to contain many virulence genes, including the type III effectors RipS5 and RipTAL. This possibly reflects a variable auxiliary gene repertoire missed during experimental studies and suggests φRSM3 may also affect evasion of plant immunity. *Myoviridae* prophages all appeared to have very similar gene contents with few auxiliary genes beyond a DNA methyltransferase, possibly explaining their lack of fitness effects in experimental studies [48, 49, 50]. However, φRSY1 contained a type IV secretion system protein and a low abundance type III effector RipS4. φRSY1 lysogens exhibit higher twitching motility and aggregation than non-lysogens [49] and φRSY1 virulence assays may have been impacted by using very low virulence RSSC strains [49]. Therefore, φRSY1 prophages may affect host fitness and should be re-examined using higher virulence strains. Novel, uncharacterised prophages also contained virulence genes with Unclassified A encoding the type III effector RipS5 and a histone-like DNA binding protein. Interestingly, phylotype IIB hosts, which predominantly harbour φRS551 and Unclassified A, typically contain an inactive, pseudogenised copy of RipS5 [82]. Previously RipS5 pseudogenisation has been attributed to disruption either by a prophage [107] or a transposon [108] due to the presence of an intragenic transposase. We found that, in the phylotype IIB strain UY031, RipS5 appears to be disrupted by an Unclassified A prophage which contains a Mu-like transposase. Therefore, prophage fitness effects may be attributed to both encoding of auxiliary gene content and host gene disruption.

Some prophage-encoded auxiliary genes were phage family or phage-type specific, including a glycosyl hydrolase and replication-associated recombination protein in *Inoviridae* RSM-type phages, and a zot-like toxin in RSS-type prophages. Moreover, *Myoviridae* prophages all encoded three glycosyl hydrolases and a DNA methyltransferase. The ubiquity of these genes suggests that they may be under strong selection, and their presence in prophages from different phylotypes indicates that selection is phylotype-independent. As these genes have all been implicated in bacterial fitness [109, 110, 111], they may have been selected for by providing general selective advantage for their hosts. Alternatively, they may provide fitness benefits to the phages themselves by encouraging phage replication and transmission. Glycosyl hydrolases have previously been linked to bacterial cell wall degradation, therefore promoting cell lysis [112], and the zot-like toxin has N-terminus similarity to the phage assembly-associated pI protein [113]. Moreover, phage-encoded DNA methyltransferases are often used by phages to evade bacterial restriction-modification systems [114]. The most prevalent prophage gene annotation was “Hypothetical protein” which represented over half of all prophage genes identified. Given low annotation power is a common occurrence in prophage studies [24, 84], we designed a Python linker tool which predicts proteins’ functions based on their Alphafold-predicted 3-D structures. The tool successfully annotated 9.4% of the most abundant hypothetical proteins reflecting a significant improvement compared to when using homology-based methods alone. Yet, these findings expose the limitations of using bacterial gene and protein databases to identify bacterial fitness-associated auxiliary genes in prophages. Therefore, where possible, future studies should combine bioinformatic auxiliary gene identification with experimental and structural analyses when assessing prophage function.

In conclusion, this study provides an insight into the global diversity and distribution of RSSC prophages at pangenome-level. Our results highlight that while RSSC prophages are highly diverse and widespread, their prevalence and distribution are proportional to their host phylotype genetic similarity. Prophages thus make a lineage-specific contribution to RSSC accessory genome, potentially affecting the fitness of their host lineages. 

## Supplementary Information

**Additional file 1:** **Figure S1.** Phylogeny of *Ralstonia solanacearum* species complex. Maximum Likelihood phylogeny was constructed based on the genomes of 192 *Ralstonia solanacearum* species complex strains from Protect and the National Collection of Plant Pathogenic Bacteria (NCPPB) and other reference strains maintained at Fera Science Ltd, along with 5 previously phylotyped and sequenced strains from NCBI Genbank (names shown at the tips of tree). Phylogenetic relationships between known phylotypes were used to assign the 192 strains sequenced in this study to given phylotype clusters. **Figure S2.** Prophage filtering and core gene detection. A) Venn diagram of putative prophages identified with PHASTER, PhiSpy, and Virsorter2 + CheckV. Prophage hits identified with multiple tools which had overlapping genome co-ordinates were clustered into groups. B) Venn diagram showing filtering steps of intact prophages identified using PHASTER: intact prophages were only kept if they were validated by an additional tool or had significant similarity to known phages in the NCBI database. **C**) Left shows a Mash tree of filtered intact prophages, similar to that in Figure 3. Right shows a heatmap of cornerstone prophage gene copy number, including “Cell lysis”, “DNA replication + packaging”, and “Structural genes”. **Figure S3.** Intact prophages have similar GC content but higher lengths than related incomplete prophages. Boxplots and violin plots of (**A**) GC content and (**B**) length of prophages. Only prophage groups with more than three intact and incomplete copies were included. Intact prophages are shown in red and incomplete prophage are shown in blue. Points are jittered to avoid overplotting. **Figure S4.** Prophage number per genome is similar between phylotypes for intact but not incomplete prophages. Boxplot and violin plot of the number of prophages per genome for isolates from each phylotype. Intact prophages are shown in red and incomplete prophages are shown in blue. Box width varies with number of isolates. **Figure S5**. Known RSSC phages cluster with prophages from the same family. Prophage neighbour-joining tree based on Mash distances. Red labels are known RSSC phages downloaded from NCBI Virus RefSeq database. Coloured bar shows prophage clusters as shown in Fig. 3.** Figure S6**. Prophages have broad geographical distributions. Bar plots showing the contribution of each prophage to the total percent of prophages in each continent. Bar charts are facetted by continent. **Figure S7**. Incomplete and questionable prophages have similar distributions to their intact copies. Maximum likelihood tree of RSSC isolates from phylotypes I, IIA, IIB, III, and IV rooted and annotated with prophage presence (dark grey), absence (white), and copy number (salmon and brickred). Coloured bars on left show phylotype clustering within RSSC tree. Coloured bars on top show prophage clusters, labelled with phage families. **Figure S8**. RSSC phylotypes have different prophages contents. Plots showing the percent of isolates in each phylotype that contain each prophage type. Phylotypes are facetted. Bars are coloured by prophage cluster. **Figure S9**. *R. solanacearum* phylotype IIB isolates have lower prophage dissimilarity and host genetic diversity than phylotypes I and IIA. Boxplots of (**A)** Average prophage dissimilarity of each phylotype, measured using average pairwise prophage Bray-Curtis distances, and (**B**) Average RSSC genetic diversity of phylotype I, IIA, and IIB isolates, measured using average pairwise Mash distances. Box width varies with sample size. **Figure S10**. RipS5 type III effector in phylotype IIB hosts may be disrupted by the novel prophage Unclassified **A. **Circular genome visualisation of *R. solanacearum* UY031 chromosome (NCBI accession: NZ_CP012687). Inner purple ring shows GC skew and black ring shows GC content. Orange ring shows bacterial coding sequences with type III effectors highlighted in red. Outer rings show the positions of Unclassified A and RS551 prophages in the chromosome.

**Additional file 2:** **Table S1.** RSSC isolates used in this study.

**Additional file 3:** **Table S2.** Reference RSSC genomes used for phylotype classification.

**Additional file 4:** **Table S3.** Predicted functions of known proteins using structure-based annotation tool.

**Additional file 5:** **Table S4A.** PHASTER prophage genome information. **Table S4B.** PhiSpy prophage genome information. **Table S4C.** Virsorter2 + CheckV prophage genome information. **Table S4D.** Combined PHASTER/PhiSpy/Virsorter2+CheckV hits with grouped prophages labels. **Table S4E.** De-duplicated prophage hits. **Table S4F.** Filtered PHASTER intact prophages with taxonomic labels and/or cross-tool validation.

**Additional file 6:** **Table S5A.** VIGA annotations of intact prophages. **Table S5B.** Megablast results of intact prophage metabolic genes (CAZy database). **Table S5C.** Megablast results of intact prophage type III effectors (Ralsto T3E database). **Table S5D.** Megablast results of intact prophage virulence factors (Phi-Base database). **Table S5E.** Predicted functions of abundant intact prophage hypothetical proteins using Structure-based function tool.

## Data Availability

The sequence data of the *Ralstonia solanacearum* strains has been deposited in the Sequence Read Archive under BioProject accession number PRJNA823737 (http://www.ncbi.nlm.nih.gov/bioproject/823737) and is scheduled for release on 29^th^ September 2022, or upon manuscript publication. The genome sequences for the integrated bacteriophages (prophages) have been deposited in GenBank (accessions in Table S4F) will be included in this BioProject record with BioProject release. Early reviewer access to the BioProject can be obtained from: https://dataview.ncbi.nlm.nih.gov/object/PRJNA823737?reviewer=4el88jub414fjq3v2d0h66khhf. R code used is available at: https://github.com/SamuelGreenrod/Prophage_MS.
