# Supplementary material for: Global diversity and distribution of prophages are lineage-specific within the Ralstonia solanacearum species complex
Source: BMC Genomics. 2022 Oct 6;23:689. doi: 10.1186/s12864-022-08909-7 (PMC9535894; doi:10.1186/s12864-022-08909-7)
Supplement: Supplementary file 1 — Additional file 1: Figure S1. Phylogeny of Ralstonia solanacearum species complex. Maximum Likelihood phylogeny was constructed based on the genomes of 192 Ralstonia solanacearum species complex strains from Protect and the National Collection of Plant Pathogenic Bacteria (NCPPB) and other reference strains maintained at Fera Science Ltd, along with 5 previously phylotyped and sequenced strains from NCBI Genbank (names shown at the tips of tree). Phylogenetic relationships between known phylotypes were used to assign the 192 strains sequenced in this study to given phylotype clusters. Figure S2. Prophage filtering and core gene detection. A) Venn diagram of putative prophages identified with PHASTER, PhiSpy, and Virsorter2 + CheckV. Prophage hits identified with multiple tools which had overlapping genome co-ordinates were clustered into groups. B) Venn diagram showing filtering steps of intact prophages identified using PHASTER: intact prophages were only kept if they were validated by an additional tool or had significant similarity to known phages in the NCBI database. C) Left shows a Mash tree of filtered intact prophages, similar to that in Figure 3. Right shows a heatmap of cornerstone prophage gene copy number, including “Cell lysis”, “DNA replication + packaging”, and “Structural genes”. Figure S3. Intact prophages have similar GC content but higher lengths than related incomplete prophages. Boxplots and violin plots of (A) GC content and (B) length of prophages. Only prophage groups with more than three intact and incomplete copies were included. Intact prophages are shown in red and incomplete prophage are shown in blue. Points are jittered to avoid overplotting. Figure S4. Prophage number per genome is similar between phylotypes for intact but not incomplete prophages. Boxplot and violin plot of the number of prophages per genome for isolates from each phylotype. Intact prophages are shown in red and incomplete prophages are shown in blue. Box width varie [file 12864_2022_8909_MOESM1_ESM.docx]

**Global diversity and distribution of prophages are lineage-specific within the *Ralstonia solanacearum* species complex**

Samuel T. E. Greenrod^1,*^, Martina Stoycheva^1^, John Elphinstone^2^, Ville-Petri Friman^1,*^

**Supplementary figures**


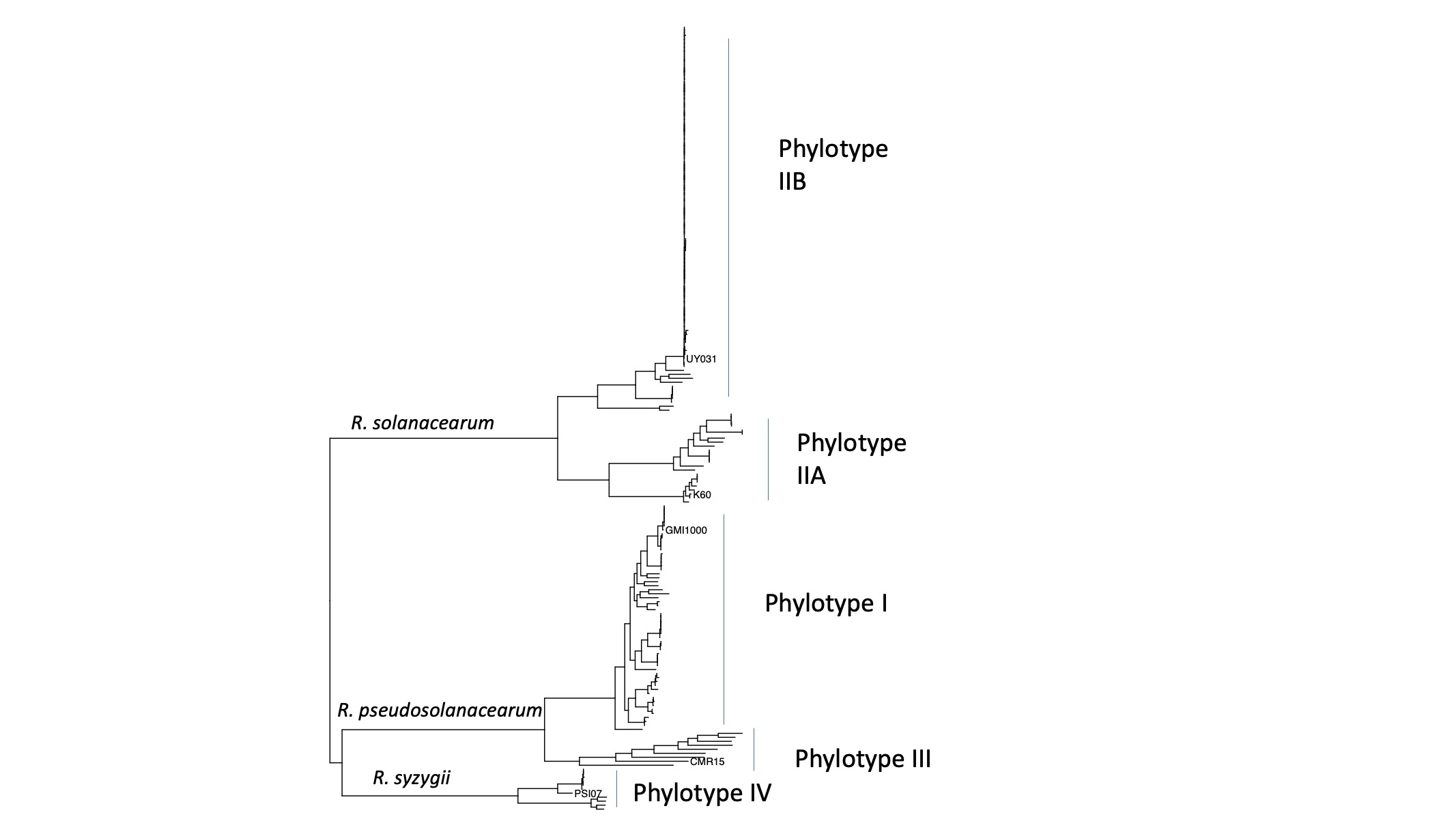


**Figure S1.** **Phylogeny of *Ralstonia solanacearum* species complex.** Maximum Likelihood phylogeny was constructed based on the genomes of 192 *Ralstonia solanacearum* species complex strains from Protect and the National Collection of Plant Pathogenic Bacteria (NCPPB) and other reference strains maintained at Fera Science Ltd, along with 5 previously phylotyped and sequenced strains from NCBI Genbank (names shown at the tips of tree). Phylogenetic relationships between known phylotypes were used to assign the 192 strains sequenced in this study to given phylotype clusters

**Figure S2.** **Prophage filtering and core gene detection.**

**A**) Venn diagram of putative prophages identified with PHASTER, PhiSpy, and Virsorter2 + CheckV. Prophage hits identified with multiple tools which had overlapping genome co-ordinates were clustered into groups. **B**) Venn diagram showing filtering steps of intact prophages identified using PHASTER: intact prophages were only kept if they were validated by an additional tool or had significant similarity to known phages in the NCBI database. **C**) Left shows a Mash tree of filtered intact prophages, similar to that in Figure 3. Right shows a heatmap of cornerstone prophage gene copy number, including “Cell lysis”, “DNA replication + packaging”, and “Structural genes”.


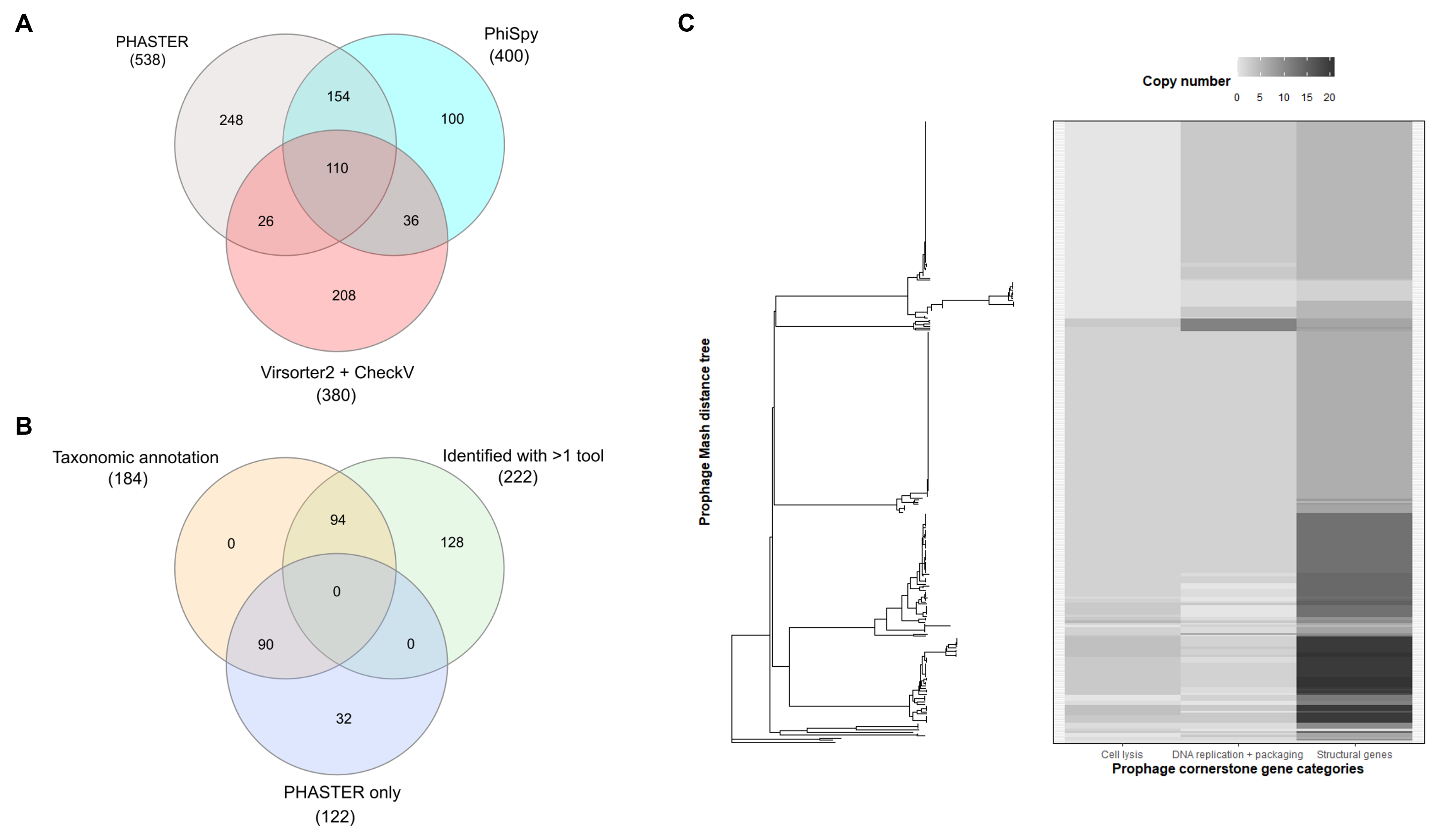


**
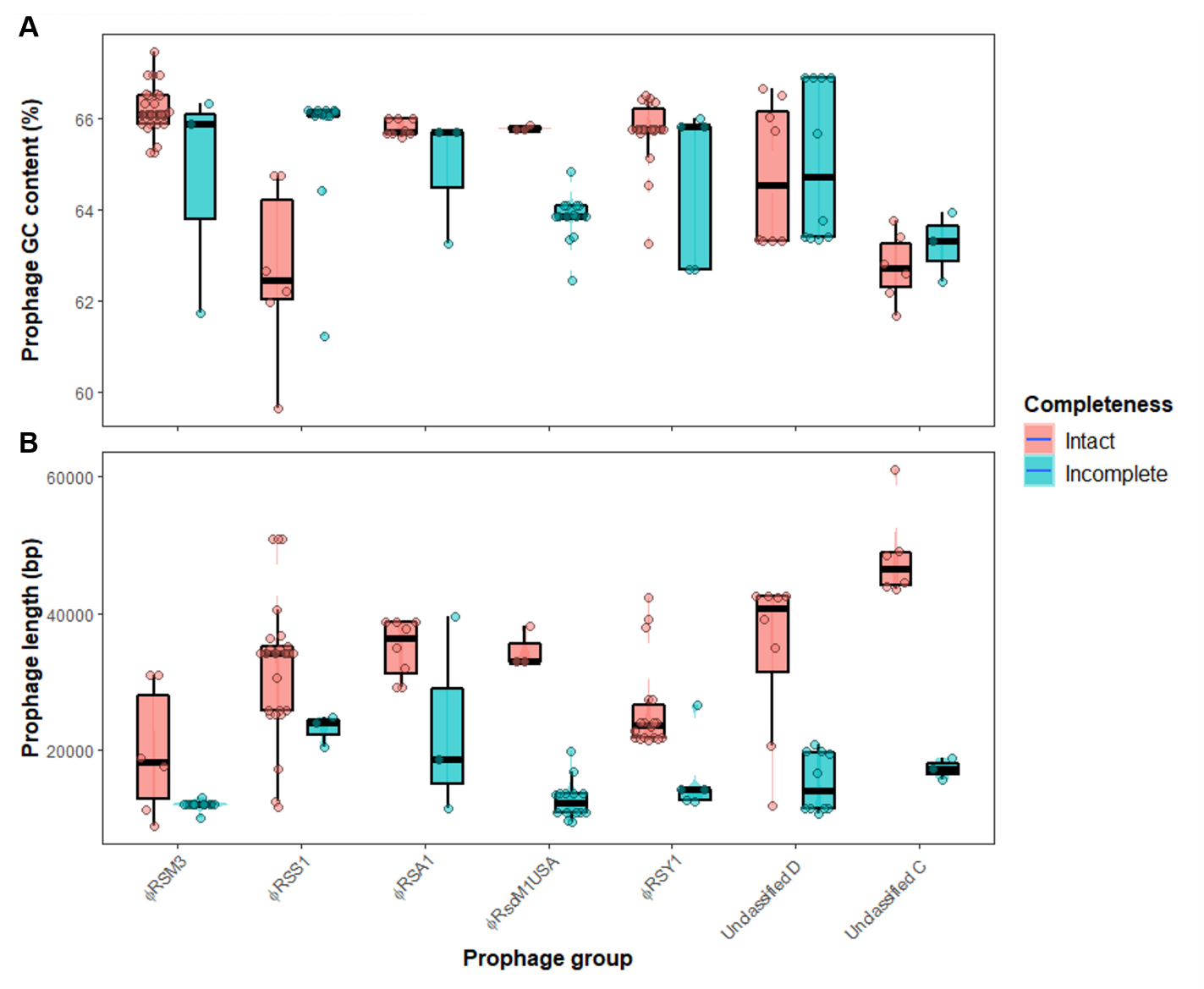
**

**Figure S3.** **Intact prophages have similar GC content but higher lengths than related incomplete prophages.**

Boxplots and violin plots of (**A**) GC content and (**B**) length of prophages. Only prophage groups with more than three intact and incomplete copies were included. Intact prophages are shown in red and incomplete prophage are shown in blue. Points are jittered to avoid overplotting.

**Figure S4.** **Prophage number per genome is similar between phylotypes for intact but not incomplete prophages.**

Boxplot and violin plot of the number of prophages per genome for isolates from each phylotype. Intact prophages are shown in red and incomplete prophages are shown in blue. Box width varies with number of isolates.


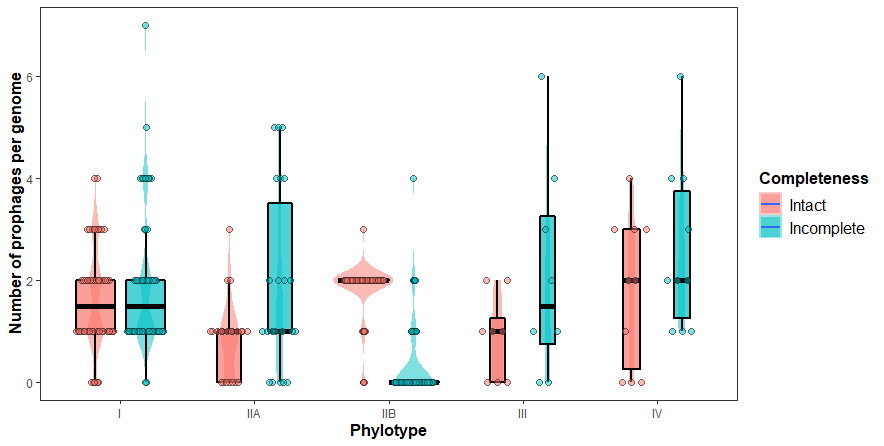


**Figure S5.** **Known RSSC phages cluster with prophages from the same family.**

Prophage neighbour-joining tree based on Mash distances. Red labels are known RSSC phages downloaded from NCBI Virus RefSeq database. Coloured bar shows prophage clusters as shown in Fig. 3.


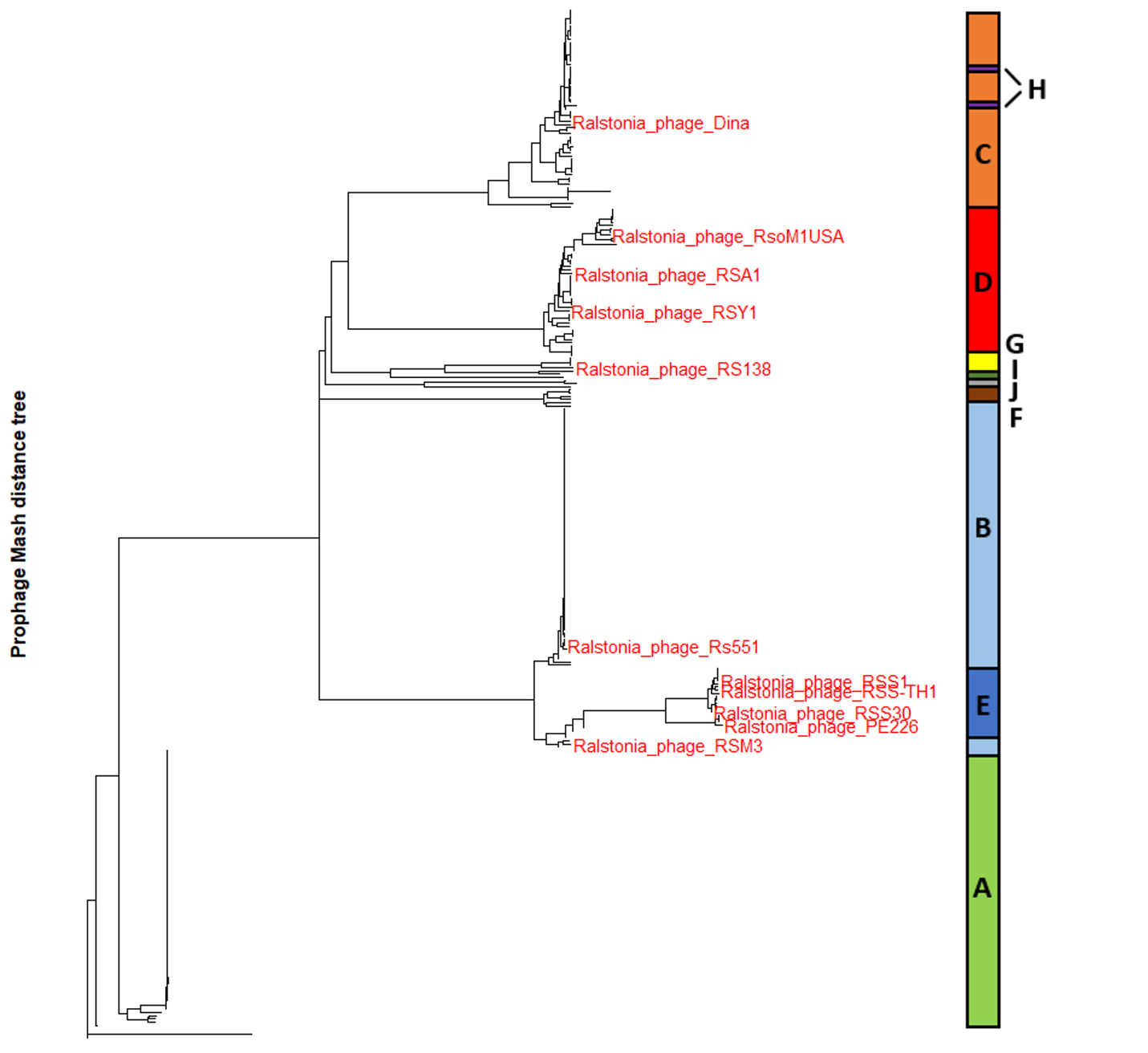

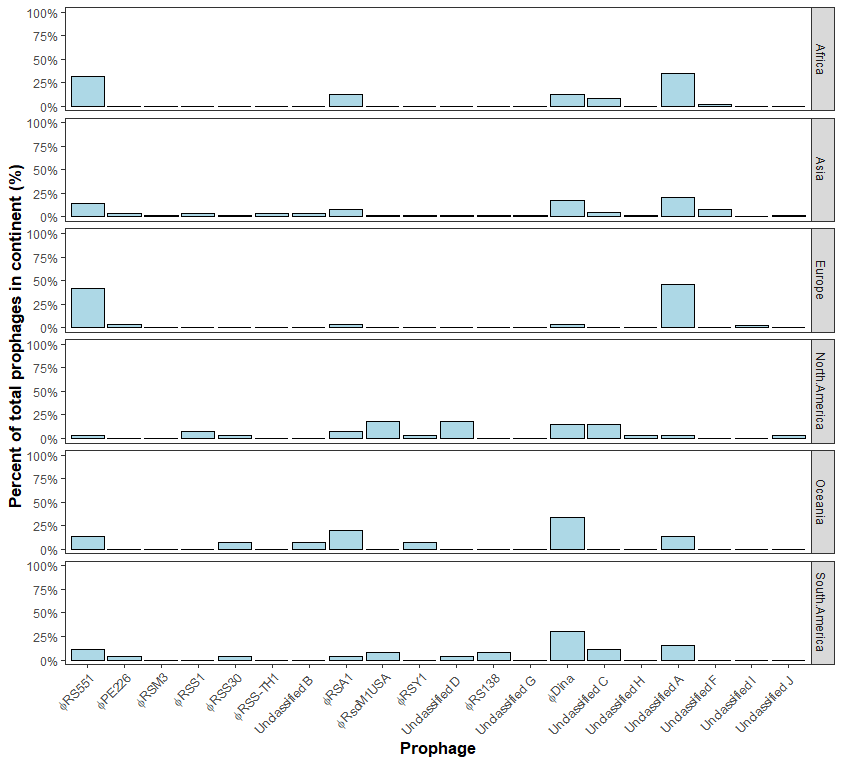


**Figure S6**. **Prophages have broad geographical distributions.**

Bar plots showing the contribution of each prophage to the total percent of prophages in each continent. Bar charts are facetted by continent.

**Figure S7**. **Incomplete and questionable prophages have similar distributions to their intact copies.**

Maximum likelihood tree of RSSC isolates from phylotypes I, IIA, IIB, III, and IV rooted and annotated with prophage presence (dark grey), absence (white), and copy number (salmon and brickred). Coloured bars on left show phylotype clustering within RSSC tree. Coloured bars on top show prophage clusters, labelled with phage families.


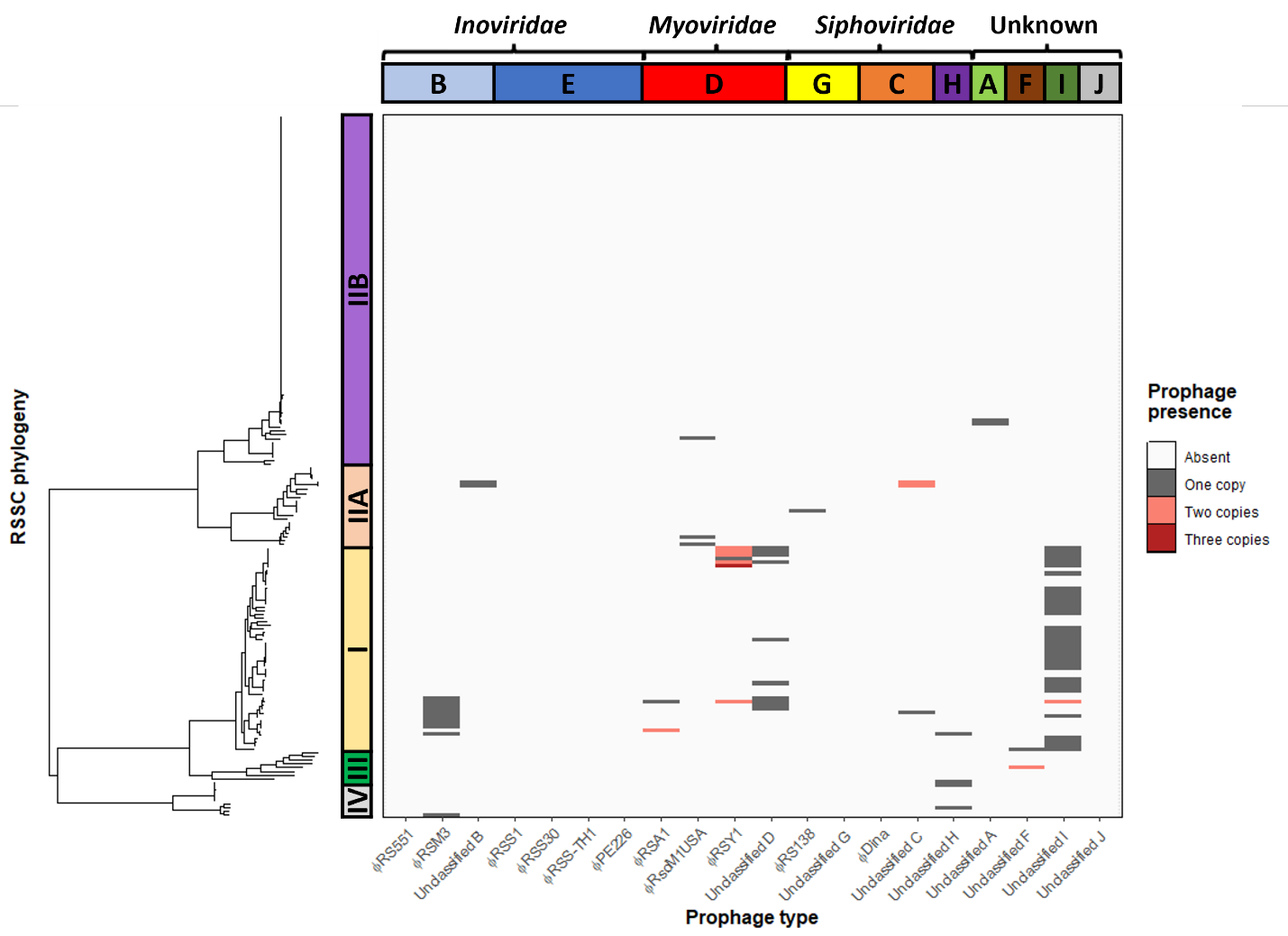


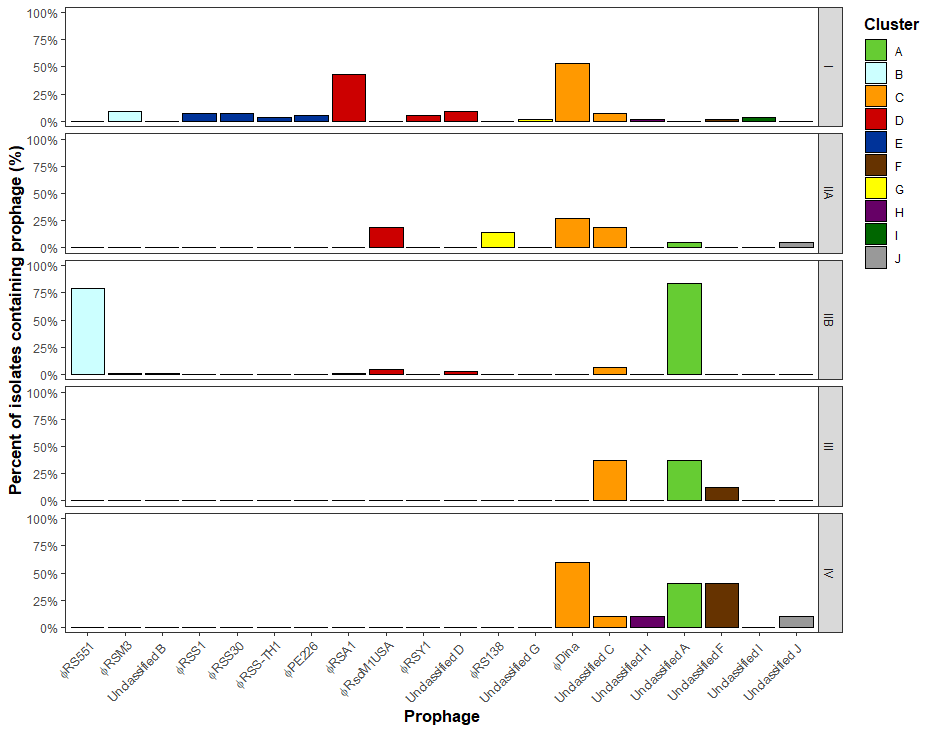


**Figure S8**. **RSSC phylotypes have different prophages contents.**

Plots showing the percent of isolates in each phylotype that contain each prophage type. Phylotypes are facetted. Bars are coloured by prophage cluster.


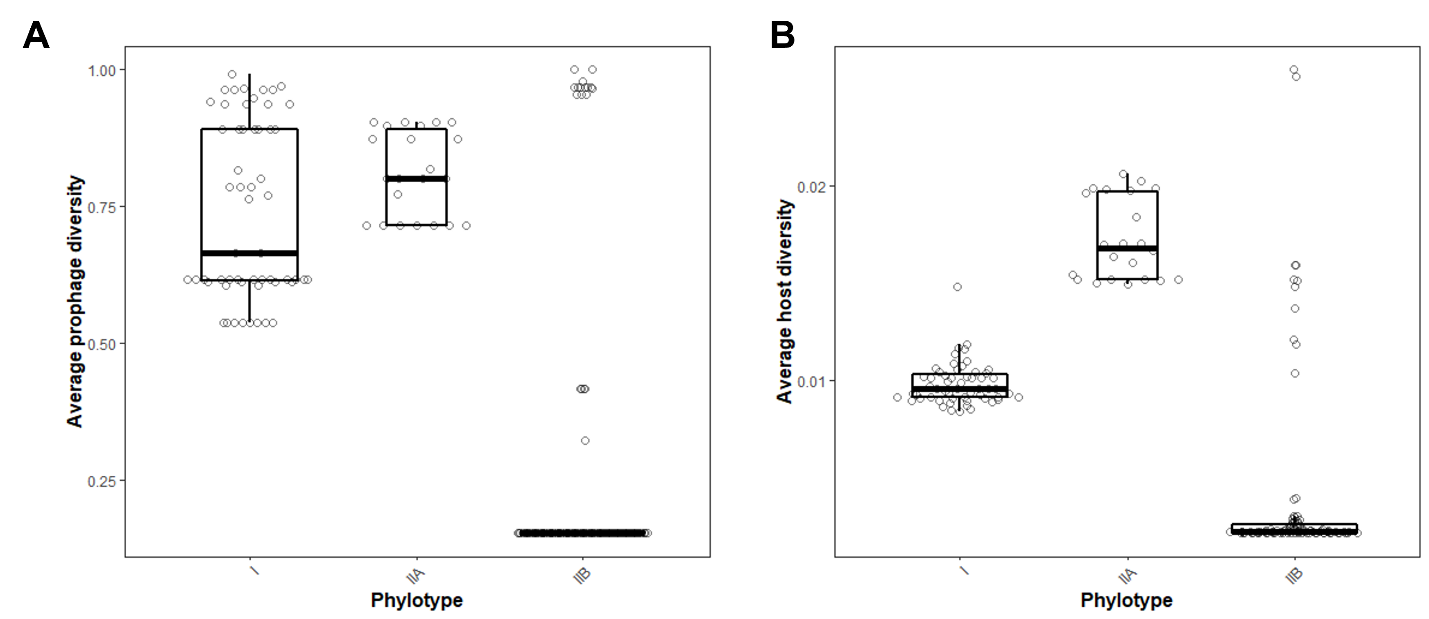


**Figure S9**. ***R. solanacearum* phylotype IIB isolates have lower prophage dissimilarity and host genetic diversity than phylotypes I and IIA.**

Boxplots of (**A)** Average prophage dissimilarity of each phylotype, measured using average pairwise prophage Bray-Curtis distances, and (**B**) Average RSSC genetic diversity of phylotype I, IIA, and IIB isolates, measured using average pairwise Mash distances. Box width varies with sample size.


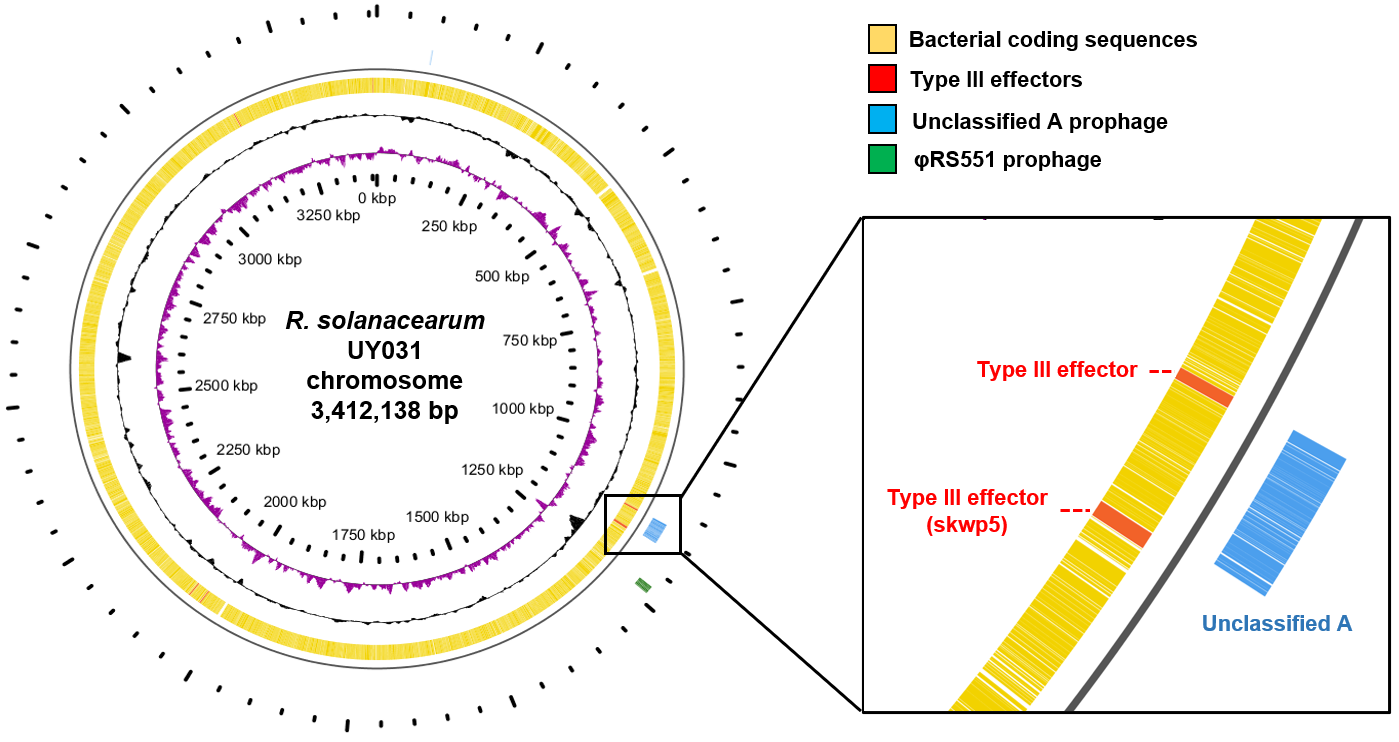


**Figure S10**. **RipS5 type III effector in** **phylotype IIB hosts may be disrupted by the novel prophage Unclassified A.**

Circular genome visualisation of *R. solanacearum* UY031 chromosome (NCBI accession: NZ_CP012687). Inner purple ring shows GC skew and black ring shows GC content. Orange ring shows bacterial coding sequences with type III effectors highlighted in red. Outer rings show the positions of Unclassified A and RS551 prophages in the chromosome.
